# Adaptive genetic potential and plasticity of trait variation in the foundation prairie grass *Andropogon gerardii* across the US Great Plains’ climate gradient: Implications for climate change and restoration

**DOI:** 10.1111/eva.13028

**Published:** 2020-06-22

**Authors:** Matthew Galliart, Sofia Sabates, Hannah Tetreault, Angel DeLaCruz, Johnny Bryant, Jake Alsdurf, Mary Knapp, Nora M. Bello, Sara G. Baer, Brian R. Maricle, David J. Gibson, Jesse Poland, Paul St Amand, Natalie Unruh, Olivia Parrish, Loretta Johnson

**Affiliations:** ^1^ Biology Kansas State University Manhattan KS USA; ^2^ State Climatologist Kansas State University Manhattan KS USA; ^3^ Statistics Kansas State University Manhattan KS USA; ^4^ Ecology and Evolutionary Biology University of Kansas Lawrence KS USA; ^5^ Department of Biological Sciences Fort Hays State University Hays KS USA; ^6^ Plant Biology and Center for Ecology Southern Illinois University Carbondale IL USA; ^7^ Plant Pathology Kansas State University Manhattan KS USA; ^8^ Hard Winter Wheat Genetics Research Unit USDA‐ARS Manhattan KS USA

**Keywords:** drought, ecotypic variation, genetic differentiation, genome–environment interaction, Great Plains grasslands, local adaptation, phenotypic variation, precipitation, reciprocal gardens

## Abstract

Plant response to climate depends on a species’ adaptive potential. To address this, we used reciprocal gardens to detect genetic and environmental plasticity effects on phenotypic variation and combined with genetic analyses. Four reciprocal garden sites were planted with three regional ecotypes of *Andropogon gerardii,* a dominant Great Plains prairie grass, using dry, mesic, and wet ecotypes originating from western KS to Illinois that span 500–1,200 mm rainfall/year. We aimed to answer: (a) What is the relative role of genetic constraints and phenotypic plasticity in controlling phenotypes? (b) When planted in the homesite, is there a trait syndrome for each ecotype? (c) How are genotypes and phenotypes structured by climate? and (d) What are implications of these results for response to climate change and use of ecotypes for restoration? Surprisingly, we did not detect consistent local adaptation. Rather, we detected co‐gradient variation primarily for most vegetative responses. All ecotypes were stunted in western KS. Eastward, the wet ecotype was increasingly robust relative to other ecotypes. In contrast, fitness showed evidence for local adaptation in wet and dry ecotypes with wet and mesic ecotypes producing little seed in western KS. Earlier flowering time in the dry ecotype suggests adaptation to end of season drought. Considering ecotype traits in homesite, the dry ecotype was characterized by reduced canopy area and diameter, short plants, and low vegetative biomass and putatively adapted to water limitation. The wet ecotype was robust, tall with high biomass, and wide leaves putatively adapted for the highly competitive, light‐limited Eastern Great Plains. Ecotype differentiation was supported by random forest classification and PCA. We detected genetic differentiation and outlier genes associated with primarily precipitation. We identified candidate gene GA1 for which allele frequency associated with plant height. Sourcing of climate adapted ecotypes should be considered for restoration.

## 1 INTRODUCTION

**FIGURE 1:**
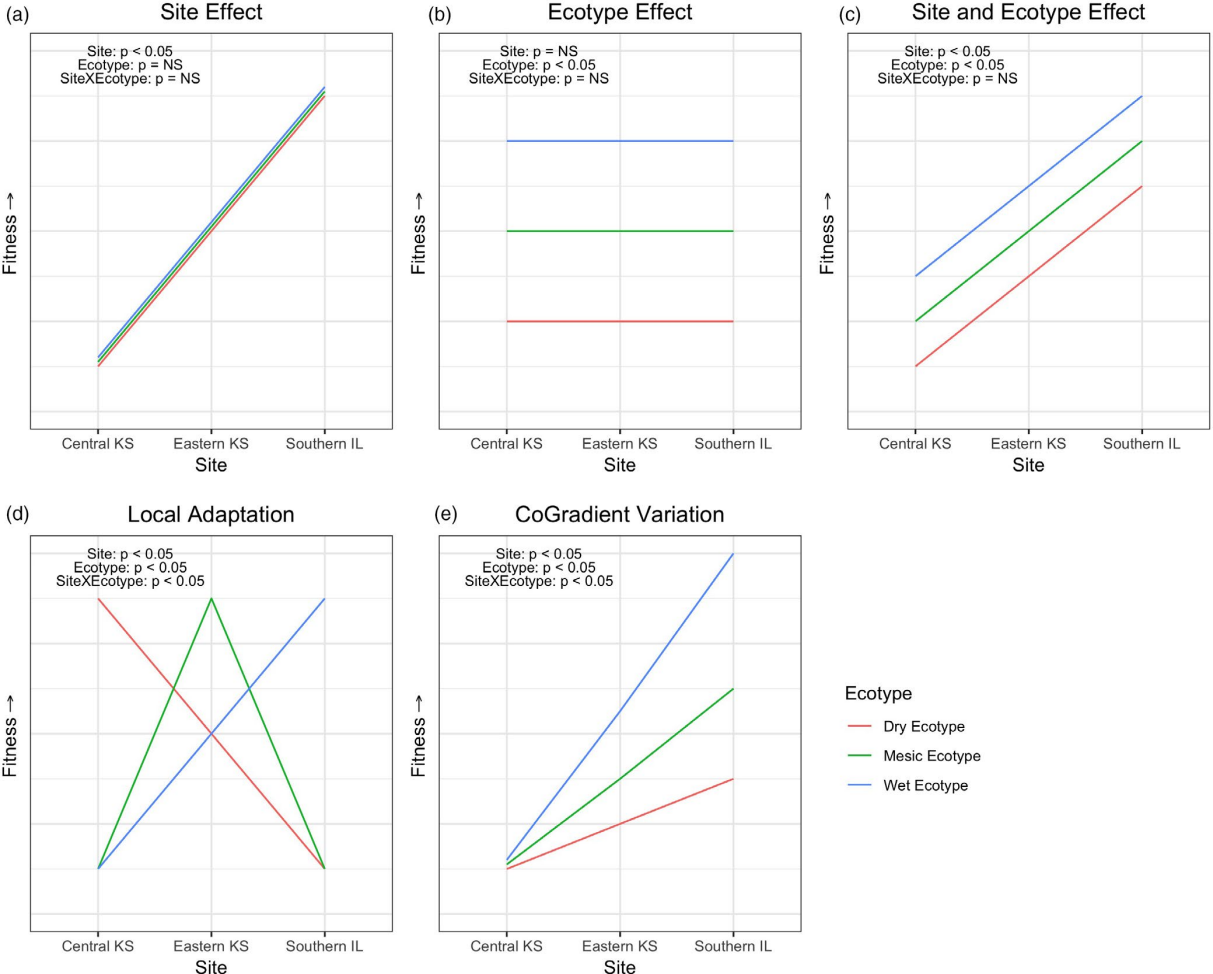
(a) Main effect of site S only, (b) main effect of ecotype E only, (c) main effects of site S and ecotype E, no interaction, (d) local adaptation S × E, (e) co‐gradient variation. Illustration of plausible phenotypic patterns of S and E effects, represented by ecotypes across an environmental gradient

Plant response to current and changing climate depends on adaptive potential within species (Des Roches et al., 2017; Etterson, 2004; Hufford & Mazer, 2003; Nicotra et al., 2010; Shaw & Etterson, 2012). More information is needed to predict how species respond to changes in climate, either through phenotypic plasticity, adaptive genetic variation or migration (Christmas, Biffin, Breed, & Lowe, 2016; Nicotra et al., 2010). Most frequently, some combination of phenotypic plasticity and genetic variation is observed in plant responses to environmental change (Conover, Duffy, & Hice, 2009; Crispo, 2008). Reciprocal gardens are a powerful approach to detect genetic variation versus phenotypic plasticity and sheds light on how species might cope with environmental change (Anderson & Gezon, 2015). If environment were the only effect on phenotype, then phenotypic variation would be entirely environmentally plastic, varying across sites yet remaining unchanged among ecotypes within a site (Figure 1a). If genotypes (we refer to as ecotype, E) were the only driver of phenotypic variation, phenotypes for each ecotype would be fixed (Figure 1b) across an environmental gradient, such that ecotype should be the same regardless of planting site. In our case, environment refers to the different planting sites (S). Another possibility is that ecotype and site both exert separate and independent main effects on phenotype (S, E, i.e., no interaction, Figure 1c). If effects of ecotype and site interact, the interaction term S × E would express the extent to which ecotypes differed in their sensitivity to environment (Figure 1d,e). One such interactive pattern is described by local adaptation, which occurs when ecotypes perform best in their home environment compared to non‐local ecotypes in the same site (Figure 1d). Finally, other forms of interaction include co‐gradient (CoGV, Figure 1e) and counter‐gradient (CnGV) variation (Anderson, Eckhart, & Geber, 2015; Chapin & Chapin, 1981; Conover & Schultz, 1995; Eckhart, Geber, & McGuire, 2004; Ensing & Eckert, 2019) whereby synergistic, positive effects (in case of CoGV) or inhibitory, negative effects (in case of CnGV) exist between environmental and genetic sources of variation. Other idiosyncratic interactions are possible as well.

In this study, we focus on phenotypic variation in ecotypes of big bluestem (*Andropogon gerardii* Vitman), a long‐lived dominant perennial and clonal C_4_ grass (Weaver & Fitzpatrick, 1932; Epstein, Lauenroth, & Burke, 1997; Knapp, Briggs, Harnett, & Collins, 1998), in response to a precipitation gradient. *Andropogon gerardii* comprises up to 80% of biomass in tallgrass prairies (Knapp et al., 1998) and has wide natural distribution across the eastern United States (http://plants.usda.gov). This species is planted widely in the 3 million ha of Conservation Reserve grassland restoration throughout the Great Plains. Within the US Great Plains, *A. gerardii* occurs along a 1,050‐km‐long precipitation gradient from western KS (500 mm of mean annual precipitation [MAP]) to Illinois (1,200 mm MAP). This precipitation gradient and these grasslands have been in place for the last 10,000 years since the last glaciation (Axelrod, 1985). This allows us to test the extent of genetic variation and phenotypic plasticity to spatially varying climate, especially precipitation, and to use the climate gradient as a proxy for future climate changes.

Grassland once covered one third of continental North America (Bailey, 1998) and 40% of Earth's surface (Gibson, 2009), and remains a threatened biome (Hoekstra, Boucher, Ricketts, & Roberts, 2005). Changes in precipitation threaten many natural ecosystems (Cook, Ault, & Smerdon, 2015; IPCC, 2017, 2018; Knapp et al., 2008; Weltzin et al., 2003). Grasslands, however, are highly responsive to precipitation change (Axelrod, 1985; Knapp, Briggs, & Koelliker, 2001). Worldwide, grasslands are characterized by frequent droughts (Craine et al., 2012; Knapp et al., 1998, 2001; Knapp & Smith, 2001), with predictions of more frequent drought for the US Great Plains in the future (Cook et al., 2015; IPCC, 2018). Yet, the degree of intraspecific variation is poorly known for most species (Des Roches et al., 2017). Furthermore, adaptive variation across environmental gradients is poorly characterized for most plants, especially for foundation plant species that largely control ecosystem processes (Whitham et al., 2006; Wymore et al., 2011). Consequently, understanding natural variation in genetic versus phenotypic plasticity across the precipitation gradient of a dominant grassland species is particularly timely in the face of climate change. Ultimately, our results will assist conservation and restoration managers to better identify the optimal climate‐matched ecotype for restorations (Kettenring, Mercer, Adams, & Hines, 2014; Pickup, Field, Rowell, & Young, 2012) and forage supply in changing climates for a major ecological foundation species (Aitken & Whitlock, 2013; Gibson, Espeland, Wagner, & Nelson, 2016; Gibson, Donatelli, AbuGhazaleh, Baer, & Johnson, 2016).

We used a reciprocal common garden approach to detect genetic and phenotypic plasticity effects on phenotypic variation of *A. gerardii* (Byars, Papst, & Hoffman, 2007; Clausen, Keck, & Hiesey, 1940; de Kort et al., 2014; Etterson, 2004; Gonzalo‐Turpin & Hazard, 2009; Lowry, Hall, Salt, & Willis, 2009; McMillan, 1959, 1965; Villemereuil, Gaggiotti, Mouterde, & Till‐Bottraud, 2016). Four reciprocal garden sites, planted with three regional ecotypes of *A. gerardii* (four populations per ecotype), span a precipitation gradient. Specifically, this study aimed to answer the following questions: (1a) How will ecotypes of *A. gerardii* respond under different climatic conditions, especially precipitation, when reciprocally transplanted into its local versus non‐local environments? (1b) More specifically, what is the relative role of genetic variation and phenotypic plasticity in controlling phenotypic differences? We hypothesized if local adaptation is strongly enforced by precipitation in the dry region, a dry ecotype would outperform foreign ecotypes when planted in the dry end of the gradient, and wet ecotypes would also show a homesite advantage in the wet end of the gradient (Figure 1d). Additionally, we planted ecotypes of *A. gerardii* outside its main distribution in the Great Plains into an even drier region of its distribution (Western KS in Colby, KS) to test the extent to which ecotypes might respond to increasingly dry conditions (Cook et al., 2015; Weltzin et al., 2003) as a surrogate for ecotypic response under future extreme dry conditions (De Frenne et al., 2013; Shaw & Etterson, 2012). (2) How do ecotypes compare when planted in their home environment? Using a subset of the reciprocal garden data with ecotypes grown in their homesite, we predicted an ecotype‐specific suite of traits such that climate drivers, especially precipitation, were expected to control morphology and fitness of ecotypes in their homesites. As precipitation became more favorable moving eastward, plants may be expected to be more robust in vegetative traits (taller, greater canopy area, wider leaves) and show increased reproductive fitness. (3) What are the underlying genetic bases for ecotype differences in traits and how are genotypes and phenotypes structured by climate? We predicted phenotypic variation is influenced by genetic differentiation among ecotypes, with genetic outliers and candidate genes potentially associated with climate, especially precipitation. (4) What are the implications for climate change and restoration? To answer these questions and test hypotheses, we present results of vegetative performance and fitness measurements of *A. gerardii* in reciprocal gardens and in their homesite garden. We further relate responses to genetic differentiation, candidate genes, and climate drivers. These results provide a comprehensive understanding of *A. gerardii* ecotype responses to climate, across the Great Plains, and will allow us to predict responses of *A. gerardii* to current and changing climates and inform restoration.

## 2 METHODS

### 2.1 Plant materials and seed collection sites

**FIGURE 2:**
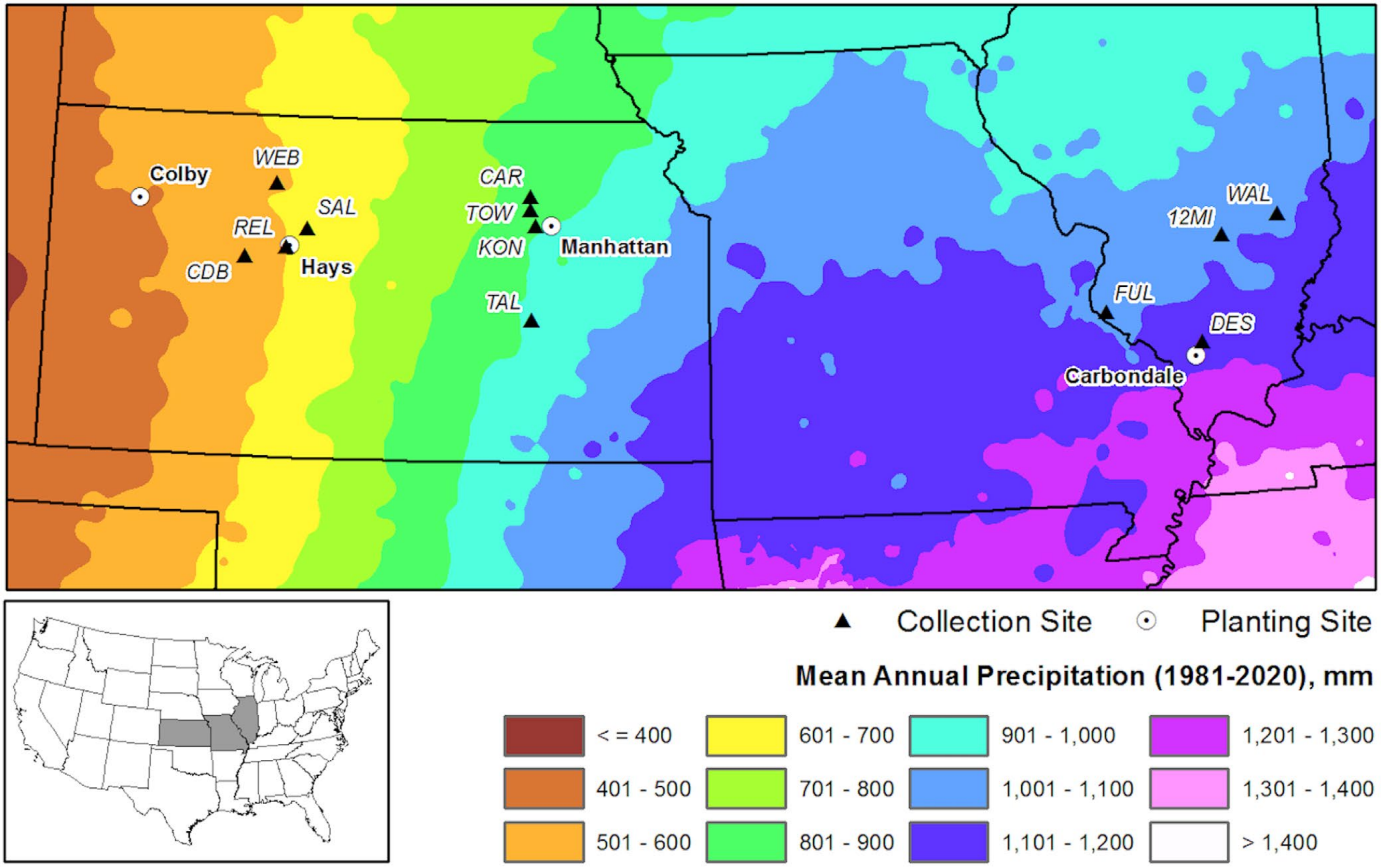
Regional map depicting the location of reciprocal gardens planting sites (white circles) and seed collections sites (black triangles) across the US Great Plains. For prairie population acronyms, see Table S1. The planting site in Western Kansas (Colby, Kansas) was the satellite reciprocal site to test the range of tolerance for big bluestem. Note that seeds were not collected in Colby

Seeds were collected by hand in autumn 2008 (three separate dates), from three climatically distinct ecoregions (Kuchler, 1964) along a precipitation gradient from central, eastern KS, and southern IL (Table 1, Figure 2): mixed grass (dry ecotype from Central KS), tallgrass (mesic ecotype from Eastern KS), and prairie savannah (wet ecotype from Illinois). Mixed and tallgrass prairies are open grassland, dominated by low stature grasses with few forbs (Knapp et al., 1998). In the prairie savannah ecoregion, diversity and structure shift to communities of tall stature, robust forbs and shrubs, and scattered trees (Kuchler, 1964). In each region, seeds were collected from four sites (Table 1, Figure 2), each referred to as a population. Populations from the same region jointly defined an ecotype. Populations originated from intact, never restored prairies generally within an 80 km radius of each reciprocal garden planting site (Table 1). Seeds were collected multiple times from each population with collections from different plants throughout each population. Several kilograms of seed were collected from each population site, so it is unlikely the plants in the plots are related. Thus, plants should be representing distinct maternal families. Seeds were stored in paper bags and kept dry at 4.4°C until germination the following spring.

**TABLE 1:** Seed collection sites defining populations within an ecotype and associated environmental information of the site. Temperature severity index is number of days over 95F/total number of days

Ecotype, Region Elevation (m)	Prairie Name (abbrev, size ha)	Lat N Long W	County, State (Weather Station)	Number of Precipitation Events > 1.25 cm (NOPPT)	Precipitation driest year (cm) (PPTDRY)	Mean annual rainfall (AMP) (cm)	Seasonal mean rainfall (SMP) (cm)	Annual diurnal temp (ADV) (°C)	Seasonal diurnal temp (SDV) (°C)	Annual mean temp (AMT) (°C)	Seasonal mean temp (°C)	Temp severity index (TS)	MAP (cm)/MAT (°C)
Dry CKS (606)	Webster Reservoir (WEB, 356)	39.41 99.50	Rooks, KS (Webster Dam)	17	25.96	58.70	39.35	15	15.4	12.4	19.8	0.100	4.72
Dry CKS (641)	Saline Experimental Range (SAL, 880)	39.02 99.14	Ellis, KS (Plainville)	16	36.32	61.7	31.0	14.6	15.5	11.8	20.7	0.088	5.22
Dry CKS (688)	Cedar Bluffs Reservoir (CDB, 850)	38.76 99.83	Trego, KS (Cedar Bluffs Dam)	16	32.18	53.31	35.97	14.4	14.5	11.2	19.5	0.091	4.72
Dry CKS (640)	Relict Prairie (REL, 14)	38.85 99.37	Ellis, KS (Hays 1S)	16	32.61	58.0	37.7	14.6	15.5	12.0	20.6	0.088	4.82
Mesic EKS (366)	Konza Prairie (KON, 1557)	39.08 96.56	Riley/Geary, KS (Manhattan 6SW)	22	68.89	88.47	56.54	12.8	12.4	12.8	21.0	0.048	6.92
Mesic EKS (92)	Tallgrass National Park (TAL, 4,409)	38.25 96.60	Chase, KS (Tallgrass Nat Park)	21	59.77	82.82	49.19	12.7	12.2	12.7	20.8	0.066	6.54
Mesic EKS (389)	Carnahan Cove (CAR, 99)	39.34 96.62	Pottawatomie, KS (Wamego)	23	52.35	87.20	53.34	13.0	13.1	13.0	21.4	0.057	6.72
Mesic EKS (379)	Top of the World Park (TOW, 61)	39.22 96.62	Riley, KS (Tuttle Dam)	21	45.11	81.12	50.70	13.1	13.2	11.7	20.3	0.057	6.95
Wet SIL (119)	Desoto Prairie (DES, 0.4)	37.85 89.14	Jackson, IL (Carbondale, Il)	33	67.41	115.92	53.53	12.3	12.6	13.2	21.1	0.027	8.78
Wet SIL (160)	Twelve Mile Prairie (12MI, 28)	38.78 88.83	Effingham, IL, (Monroe, Fayette, Salem)	25	70.01	107.57	51.83	11.7	12.4	12.3	18.7	0.026	8.72
Wet SIL (150)	Walters Prairie (WAL, 5)	38.92 88.19	Jasper, IL (Newton/ Charleston)	27	69.18	104.04	50.80	10.8	11.7	13.4	21.8	0.014	8.11
Wet SIL (215)	Fults Prairie (FUL, 214)	38.17 90.19	Monroe, IL (Sparta)	31	69.38	111.27	55.14	11.9	12.6	13.2	21.0	0.031	8.38

Note

Number of precipitation events > 1.25 cm per year. Soils were mostly loam as follows: Wakeeney‐Harney Silt loam (WEB), Bogue‐Armo Clay loam (SAL) ArmoClay loam (CDB) Armo loam and Brownell gravelly loam (REL), Benfield Florence silty clay loam (KON), Cline Sogen Silty clay loam (TAL), Benfield Florence silty clay loam (CAR), Irwin silty clay loam (TOW), Orthents silty loam (DES), Cisne silty loam (12MI), Atlas silty clay loam (WAL), and Menfro silty clay loam (FUL).

### 2.2 Reciprocal garden planting sites

Reciprocal garden plants were used to measure vegetative and reproductive morphology of ecotypes in relation to climate and to characterize genetic diversity, structure, and outlier loci. Seeds collected from native prairie were used in the reciprocal gardens. In winter 2009, a subset of seeds from each population was germinated and grown in greenhouse using potting mix (Metro‐Mix 510). In August 2009, 3‐ to 4‐month‐old plants of all 12 populations were planted at each of four garden sites: western KS in Colby, central KS in Hays, eastern KS in Manhattan, and Illinois in Carbondale (Figure 2, Table 2). Phenotypic measurements began 2 years after germination and planting in the gardens, thus minimizing maternal effects and transplant shock. At each planting site, the design consisted of 10 rows (blocks), each containing 12 plants corresponding to 4 four populations of each of the three ecotypes in random order (10 blocks × 12 plants (4 populations × 3 ecotypes) = 120 plants per site) (Table 1, Figure 2). Although populations of *A. gerardii* can be found at the western range of distribution in western KS, by design, there was no western KS ecotype (from Colby). The goal for this study was to assess the ecotypes found within the core of *A. gerardii* distribution. However, the western KS planting site was incorporated into the reciprocal garden to test tolerance of ecotypes to even drier conditions, similar to what *A. gerardii* might experience predicted by climate change. These very dry western KS prairies are similar physiognomically to the other grasslands, with similar clay loam soils, and similar species composition. (Unfortunately, in all of these sites, we cannot control for any differences in biotic community such as herbivores and microbes). Within each row, plants were spaced 50 cm apart. The soil around each plant was covered with water‐penetrable landscape cloth to discourage growth of competing plants. Nearly all plants of all ecotypes survived transplantation to all sites, even into western KS. Note that these reciprocal gardens with single‐spaced plants of only *A. gerardii* are a separate research platform (described Caudle, Johnson, Baer, & Maricle, 2014; Kramer et al., 2018; Maricle, Caudle, Lindsey, Baer, & Johnson, 2017; Mendola, Baer, Johnson, & Maricle, 2015; Olsen, Caudle, Johnson, Baer, & Maricle, 2013; Varvel et al., 2018) from reciprocal gardens with *A. gerardii* ecotypes planted with other prairie species in a seeded community (Galliart et al., 2019; Johnson et al., 2015; Maricle et al., 2017; Wilson, Gibson, Baer, & Johnson, 2016).

**TABLE 2:** Geographical descriptors and summary of historical weather data (30‐year normals) as descriptors of environmental conditions for the planting sites of the reciprocal garden platform

Reciprocal garden planting site (Town, County) Soil Type	Elev. (m)	Lat. (°N) Long (W)	Rainfall 6‐year mean 2006–2009 (range) (cm)	Annual number of Pcp events >1.25 cm (NOPPT)	Pcp driest year (cm) (PPT DRY)	Mean annual rainfall (cm) MAP	Growing season mean rainfall (cm) (sum + sp)	Annual diurnal temp (°C)	Growing seasonal diurnal temp (°C) (sum + sp)	Annual mean temp (°C)	Growing Season mean temp (°C) (sum + sp)	Temp severity index (# days over 95F)
Western KS (Colby, KS Thomas, Co) KSU Ag Expt Station (Ulysses Silt Loam)	972	39.39 101.06	48.0 (29.4–66.8)	13.0	28.37 (1967)	52.5	39.44	−2.0	−2.0	10.9	16.7	21.3
Central KS (Hays KS Ellis Co) KSU Ag Expt Station (McCook Silt Loam)	603	38.85 99.34	54.6 (38.3–67.9)	15.4	36.27 (1988)	59.6	43.18	−3.2	−3.4	12.3	18.3	29.2
Eastern KS (Manhattan, KS Riley Co) USDA plant materials (Belvue Silt Loam)	315	39.19 96.58	89.1 (61.5–110.2)	21.9	39.16 (1966)	90.5	63.47	−4.2	−4.3	12.8	18.9	23
Southern Illinois (Carbondale IL Jackson, Co) SIU Ag Research Station (Stoy Silt Loam)	127	37.73 89.17	125.6 (76.2–173.8)	32.7	67.38 (1963)	119.8	64.51	−5.3	−5.1	13.5	19.0	6.3

### 2.3 Climate and soil

The four garden planting sites (Table 2) were all under agricultural cultivation prior to reciprocal garden establishment and were characterized as silt loam soils (Mendola et al., 2015). For each planting site, data on long‐term average rainfall and temperature were collected from local agricultural research stations or nearby NOAA weather stations. Climate information from the population source of origin (where seeds were collected) was gathered from nearby NOAA stations (Table 1).

### 2.4 Phenotype response variables

Phenotypic measurements began in 2010, two years after germination and planting in the gardens, thus minimizing maternal effects and transplant shock. In 2010, we made non‐destructive vegetative measurements of all plants in all sites. Measurements were made during the height of the growing season (maximum biomass, mid‐summer). Once plants were firmly established by 2011, we made a destructive collection of biomass at the end of the growing season in September/October 2011. In 2012, we mainly measured reproductive responses during end (August/September) of the growing season. Some variables (canopy area, diameter, height) were measured in 2010 and 2014, 2 and 5 years post‐transplant, to examine interannual variation. While we would have preferred to measure all traits in all years, this would have been very difficult as these sites are 1,000 km apart. Therefore, for those traits that we repeated in another year, we chose to measure traits that would reflect the overall plant status such as canopy area, height, and diameter. We did not harvest roots because of its destructive nature and harm to long‐term plots.

#### 2.4.1 Vegetative response variables

##### Vegetative emergence

First emergence from the ground was recorded starting in March, and plants were observed once per week to detect first emergence. Time of emergence was expressed in Julian days and recorded as the difference from the earliest Julian day of emergence observed in the dataset.

##### Canopy area

Non‐destructive estimates of canopy area were made using photographs of all plants at all garden sites during July in 2010 and 2014. Images were taken using a Nikon Coolpix camera from directly above each plant with white background. A ruler was placed next to the plant to set image scale. Images were imported into ImageJ v1.8.0 and converted to black and white to delineate plant from background. Canopy area was determined by pixel counts (Image J, Rasband 1997–2008, online resource‐https://imagej.nih.gov/ij/) after selecting outline of plant and using a reference scale to provide area in cm^2^. Measurement error was approximately 2% based on repeated measurements of the same ImageJ photographs. On a separate set of non‐study plants, we correlated ImageJ canopy area with actual leaf area measurements (the gold standard for leaf area) from plants using a leaf scanner (Figure S2, *r* = .95 *p* < .0001).

##### Plant canopy diameter

The images taken for canopy area were also used to determine plant canopy diameter, defined as two orthogonal measurements of plant width (cm); these measurements were subsequently averaged. Measurements were made in 2010 and 2014.

##### Height

Plant height was measured in 2010 and 2014. Height was determined by extending leaves vertically and measured to the nearest cm. Measurement were taken from the ground to the highest point of extension. Reproductive stalks were not included.

##### Blade width

On each plant, two mature leaves were measured in mm at their broadest section (at approximately 2/3 from the tip of the leaf) and recorded to nearest full unit; measurements were then averaged for each plant. Blade width was measured in 2010.

#### 2.4.2 Vegetative and reproductive biomass

We harvested reproductive and vegetative biomass by cutting the plant at soil level, storing in bags for drying, and later separation and weighing. Vegetative biomass included all leaves, while reproductive biomass included flowering stalks and seeds. All samples were weighed on a Denver Instruments balance DI‐5K or Ohaus Precision standard balance. Results are presented as grams per plant.

#### 2.4.3 Reproductive characteristics

##### Anthesis

Anthesis was defined as occurring when anthers were first visible. Data are presented for 2012. In each site, plants were observed twice per week. For each plant, a binary flowering response was recorded (yes flowering = 1; no flowering = 0). When anthesis was observed, days to anthesis relative to emergence were also recorded.

##### Seed production

Seed and stalks were collected in 2012. Dates of collections occurred multiple times from September to November to ensure all seeds were collected. For each plant, a binary seed production response was recorded (yes seed production = 1; no seed production = 0). For those plants producing seed, all seeds were stored in paper bags and air‐dried, seeds and stalk were then separated by hand and each was weighed in grams.

#### 2.4.4 Statistical analyses of phenotype response variables

For phenotype responses with one measurement year (vegetative emergence, blade width, biomass, days to anthesis, probability of anthesis, seed production, and probability of seed production), statistical analyses were conducted using a generalized linear mixed model fitted to each variable with a probability distribution that recognized its continuous or discrete nature, accordingly. The linear predictor included fixed effects of planting site (western KS, central KS, eastern KS, and Illinois), ecotype (dry, mesic, wet), and their 2‐way interaction. The random effects of block nested within planting site and population nested within ecotypes were fitted to recognize the experimental units for planting sites and ecotypes, respectively. By fitting a population effect, the model explicitly incorporates variation between populations within an ecotype. Models for all traits were fitted using the GLMMIX procedure of SAS v9.3. For all traits, the heterogeneous residual variances were fitted for each planting site to enhance model fit, using maximum likelihood‐based Bayesian information criteria. Multiple testing for all traits was subjected to Bonferroni adjustments to prevent inflation of type I error. Least square mean estimates and estimated standard errors are presented. Special statistical considerations are described below.

For vegetative emergence, the model assumed a Poisson distribution of the response implemented with a log link function to account for the integer count nature of the response. The likelihood‐based Pearson chi‐square/*df* statistic did not indicate any evidence of overdispersion. Parameter estimation was conducted using residual pseudolikelihood with Newton‐Raphson with ridging as the optimization technique. Kenward–Roger's procedure was used to estimate degrees of freedom and to make the corresponding adjustments in estimation of standard errors.

For anthesis, the statistical model contained missing cells, which corresponded to the wet and mesic ecotypes in the Colby planting site, as no data were available because plants of these ecotypes did not reach anthesis in Colby. Thus, inference is limited to the combinations of ecotype and site for which data were available. Model assumptions were checked using studentized residuals and were considered to be reasonably met. Kenward–Roger's procedure was used to estimate degrees of freedom and adjust estimates of standard errors. For probability of anthesis, overdispersion was evaluated using the maximum likelihood‐based fit statistic Pearson chi‐square/DF. No evidence for overdispersion was apparent. The final statistical model used for inference was fitted using residual pseudolikelihood. For seed production, model assumptions were checked using studentized residuals and were considered to be appropriately met. Kenward–Roger's procedure was used to estimate degrees of freedom and adjust estimates of standard errors. Estimated least square means for levels of the fixed effects of interest are reported after backtransformation to the original data scale.

For traits measured in multiple years, in addition to the fixed effects of ecotype, site and ecotype*site, the fixed effect of time and their 3‐way interaction was included to account for repeated measures across the two collection years. This 3‐way interaction was included for all traits measured in multiple years (canopy area, diameter, and height). Model assumptions were checked using studentized residuals and were considered to be appropriately met. Models for all traits were fitted using the GLMMIX procedure of SAS v9.3. For all traits, the heterogeneous residual variances were fitted for each planting site to enhance model fit, as determined using maximum likelihood‐based Bayesian information criteria. Least square mean estimates and estimated standard errors are presented. In addition, for canopy area, a general linear mixed model was fitted to the response "canopy," expressed in the log scale. Kenward–Roger's procedure was used to estimate degrees of freedom and make the corresponding adjustments in estimated standard errors. Least square mean estimates expressed in the original scale after backtransformation are presented.

#### 2.4.5 Differential canopy area response of ecotypes to rainfall

Comparisons of canopy area to difference between rainfall of population of origin and rainfall from planting site were conducted in SAS v9.3 using the GLMMIX procedure. For differential canopy area in response to rainfall, we used a quadratic function (*R* ^2^ = .97 for all ecotypes) because it fit the data better than a linear function (Dry ‐ *R* ^2^ = .812, Mesic – 0.84, Wet – 0.85). The model included the fixed effects of ecotype, difference in rainfall, and difference in rainfall^2^ and the random effects of population nested within ecotype and blocks nested within site. The slopes of ecotype response versus rainfall differential were compared using an ANCOVA to identify whether the responses to rainfall differs between ecotypes, that is, do certain ecotypes exhibit a greater increase in canopy area with increasing rainfall.

### 2.5 Comparison of ecotypes in their home environment

In order to characterize whether each ecotype had a distinct suite of phenotypic traits, we compared phenotype variables of each ecotype in their homesite, namely dry ecotype in central Kansas, mesic ecotype in Eastern Kansas and wet ecotype in Illinois. (Note that we did not use western KS plants because we did not have a western KS home ecotype.) We used 106 plants comprising dry (34), mesic (35), and wet ecotypes (37). We also used random forest classification and PCA as complementary approaches to characterize ecotypes in home environment.

Random forests were used to test classification of ecotypes based on morphological traits in homesite (Breiman, 2001). Random forests use an ensemble method (Altman & Krzywinski, 2017) for classification based on morphological traits and operates by constructing many decision trees at training and taking a weighted vote of predictions from these trees for final prediction, in our case, ecotype. Implementation of random forests and description of cross validation approach, and classification error are presented in Appendix S1.

For PCA, a data subset consisted of seven traits (i.e., canopy area, height, blade width, diameter and seed production, days to emergence, and days to anthesis). Plants that did not flower were included with date of flowering one week past the last flowering date observed. Each variable was standardized to a zero mean and a variance = 1 due to differential scaling. Scree plots were evaluated to describe the proportion of the total variance described by each principal component. Data were analyzed using PCA as implemented in R v2.15.3. A stepwise model selection approach was used to explore associations between the first three PC scores and (Table 1) environmental explanatory variables. The Schwarz Bayesian information criterion was used as criterion for model fit and selection. Stepwise selection was conducted using the GLMSELECT procedure of SAS v9.3.

### 2.6 Genotyping and genetic analyses

The objective was to characterize genetic diversity among ecotypes, identify genetic outliers and candidate genes among SNPs, relate genetic outliers to climate of population source of origin, and relate genotype to phenotype in homesite. We used genotyping‐by‐sequencing to identify SNPs (Elshire et al., 2011; Lu et al., 2013; Narum, Buerkle, Davey, Miller, & Hohenlohe, 2013). Leaf samples were collected in 2014 from the same plants as used for phenotyping within the reciprocal gardens from the central KS, western KS, and southern IL planting sites. Total number of plants genotyped (314) included dry (110), mesic (106), and wet (98). DNA sample collection, preparation, and SNP calling analyses can be found in Appendix S1.

#### 2.6.1 Genetic differentiation

Pairwise population differentiation F_ST_ was implemented in *GenAlEx* v6.503 (Peakall & Smouse, 2006, 2012) using twelve populations comprising three regional ecotypes. PCoA of genetic distance was calculated from SNP markers performed in R package *Adgenet* (Jombart, 2008). Scores for genetic distance principal coordinates 1, 2, and 3 were each regressed on 11 environmental explanatory variables (Table 1) using stepwise model selection. For the PCoA stepwise regression using climate variables, we used Schwartz Bayesian information criterion to identify which environmental variables from population of origin are most associated with genetic divergence. Stepwise regression was implemented using GLMSELECT procedure in SAS v9.3.

#### 2.6.2 Outlier genetic analysis and association with climate variables

We identified “outlier” SNPs as those that show greater differentiation compared to background using two independent methods, *Bayescan* and *Bayenv* and related their differentiation to population climate of origin. We used a Bayesian approach to estimate the posterior probability that a marker is under selection as implemented in *Bayescan v2.1* (Foll & Gaggiotti, 2008) to identify SNP outliers (Lotterhos & Whitlock, 2015). To relate climate variables of population climate of origin (Table 1), we evaluated strength of association between outlier F_ST_ and 11 environmental variables using *BayeScEnv* (Villemereuil & Gaggiotti, 2015).

Second, we used *Bayenv2*, a robust approach that provides correction for population structure and demographic processes while controlling false positives (Guenther & Coop, 2013; Lotterhos & Whitlock, 2015). Population differentiation ranking statistic X^T^X (Guenther & Coop, 2013) was calculated for all loci to identify loci with greater differentiation than under neutral drift among populations. See Appendix S1.

Lastly, partial redundancy analyses (pRDA) were used to estimate the role of geographic differences (latitude and longitude) versus climate (Table 1) in structuring genetic variation. pRDA is an ordination technique (Oksanen *et al*. 2015) that partitions variation, in our case genetic variation, due to climate and geography and joint contribution of climate and geography (Riordan *et al*. 2016). See Appendix S1.

#### 2.6.3 Relating genotype to phenotype

We performed separate genome wide association (GWAS) of the 4,641 SNP markers with each of the phenotypic variables measured, namely emergence, canopy area, height, blade width, diameter, seed weight, and days to anthesis, using *TASSEL* v5.0 software (Bradbury et al., 2007). Association in *TASSEL* v5.0 was performed using a mixed linear model, including a kinship matrix to account for relatedness between individuals along with Q values from *Structure* v2.3.4 (Falush, Stephens, & Pritchard, 2007) to account for population structure. Run parameters for STRUCTURE included 20,000 burn‐in and 500,000 MCMC chain length. Admixture was included, and correlation between alleles was not assumed. Three separate iterations per K were performed. To identify optimal number of *K* genetic clusters, Evanno's delta *K* was calculated in *Structure Harvester* v0.6.94. *K* clustering and permutation were done in *CLUMPP* v1.1.2. SNPs were individually associated with phenotype, and Bonferroni multiple test correction was used to identify SNPs significantly associated with phenotypes. Data consisted of plants from their home planting site only (106 plants total, 34 dry ecotype in Central Kansas, 35 mesic ecotype in Eastern Kansas, and 37 wet ecotype in Illinois). Unfortunately, we cannot present Manhattan plots due to the lack of availability of a genome for *A. gerardii*.

## 3 RESULTS

### 3.1 Reciprocal gardens show pattern of phenotypic plasticity and ecotype genetic effects on phenotypic variation

#### 3.1.1 Vegetative responses

##### Date of vegetative emergence

**FIGURE 3:**
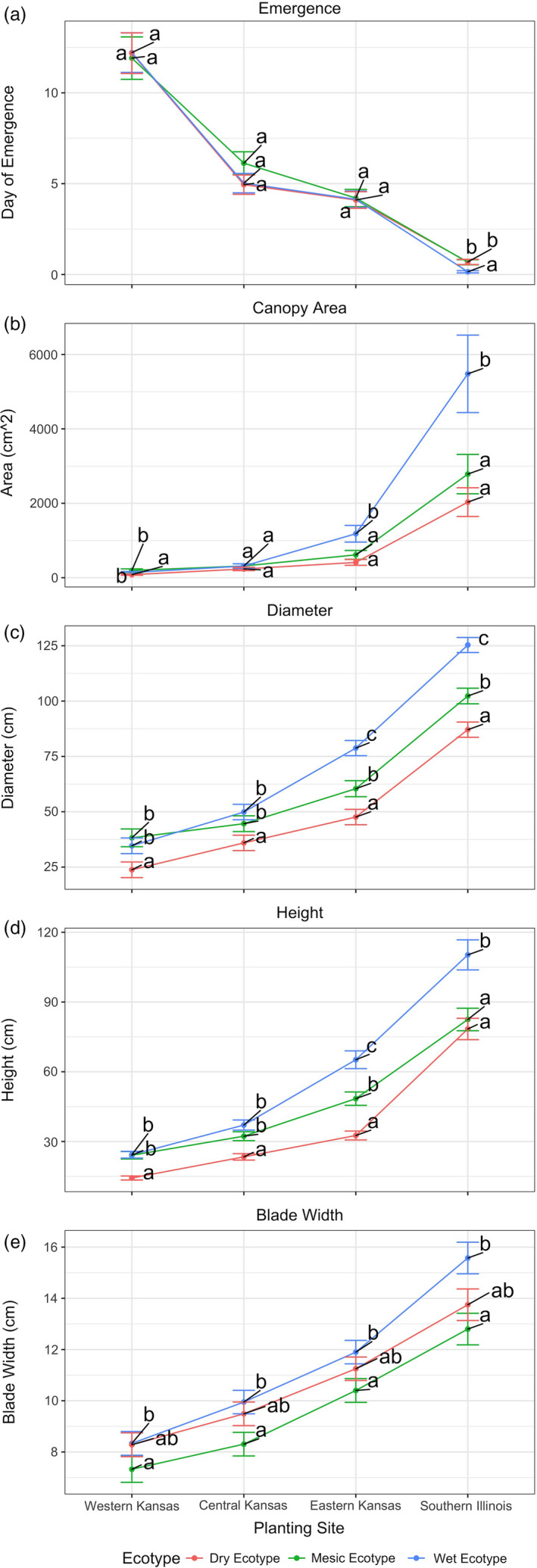
Least square mean estimates (±*SE*) of vegetative morphological traits for ecotypes (dry, mesic, wet) across reciprocal garden planting sites in Western Ks (Colby KS), Central KS (Hays KS), Eastern KS (Manhattan KS), and Illinois (Carbondale Illinois). (a) Days at emergence, (b) canopy area (cm^2^), (c) plant diameter (cm), (d) plant height (cm), and (e) blade width (mm). Sites with different letters indicate significant differences within a site. Biomass is included in Figure S4

Given significant interaction between site (S) and ecotype (E) (*p* = .0107, Figure 3a, Tables S1 and S2), we focused inference on simple effects. That is, we conduct pairwise comparisons between sites for a given ecotype and second, between ecotypes within a given site. Overall, the most striking pattern in time to emergence (relative to first day of emergence) was a general decrease in ranking of days to emergence Weatern KS > Central KS > Eastern KS > Illinois with emergence occurring 12 days later in the westernmost sites relative to the easternmost site. Comparing ecotypes within a site, for all ecotypes, there was no evidence for differences in time to emergence for all KS sites. However, in Illinois, the wet ecotype emerged slightly earlier (half day) than other ecotypes.

##### Canopy area

Canopy area showed a significant 3‐way interaction between S, E, and Time (T) (*p* = .0174, Table S2). To explain this 3‐way interaction, we conduct simple‐effects analyses for each year (Figure 3b and for 2014, Figure S3). Overall, the general pattern of canopy area for 2010 and 2014 seems to be consistent with CoGV (Figure 1e), with an ecotype‐specific increase in canopy area from west to east. Specifically, at the dry end (i.e., western KS and central KS sites), all ecotypes showed small canopy area and only small differences between ecotypes were detected. Moving east toward more favorable climates, ecotypes showed increasing canopy area and maintained their relative rankings (i.e., dry < mesic < wet ecotypes) though differences between ecotypes increased toward the east. For example, the dry ecotype canopy area increased from western KS site (82.6 ± 15.8 cm^2^) to Illinois (2,031.8 ± 286.0 cm^2^) while in the same locations, the wet ecotype increased from 140.2 (± 26.9) cm^2^ to 5,479.2 (± 1,040.8) cm^2^. Furthermore, the wet ecotype showed a disproportionately larger canopy relative to dry and mesic ecotypes in the two wetter‐most sites (i.e., eastern KS and Illinois). A similar general pattern holds for 2014 (Table S1, Figure S3a), except ecotype‐specific canopy areas across sites were substantially larger, probably due to the fact that plants were bigger in 2014 because they were more established.

##### Canopy diameter

We measured canopy diameter in 2010 and 2014. Canopy diameter showed a significant 3‐way interaction between S, E, and T (*p* = .0002, Table S2). To explain this 3‐way interaction, we conduct simple‐effects analyses for each year. For brevity, we compare ecotypes within each site. Figure 3c shows estimated mean canopy diameter (±*SE*) for ecotypes at each site in 2010 and 2014 (Figure S3). The general pattern of canopy diameter for 2010 and 2014 appears consistent with CoGV (Figure 1e), with an ecotype‐specific increase in canopy diameter from west to more favorable climates of the eastern sites (Table S1). Specifically, at the dry end (i.e., western KS and central KS sites), all ecotypes showed small canopy diameter with only small, but significant, differences between ecotypes detected. The dry ecotype had significantly smaller diameter than mesic and wet ecotypes at the dry end. Moving east on the gradient, ecotypes showed increasing canopy diameter, increasing by as much as 3.6‐fold, while maintaining their relative rankings (i.e., wet > mesic > dry ecotype) though differences in diameter between ecotypes increased toward the east, similar to canopy area. For eastern KS and Illinois sites, all ecotypes were significantly different from each other within a site with ranking wet > mesic > dry with the wet ecotype increasing diameter disproportionately. A similar general pattern holds for 2014 (Table S1, Figure S3b), except ecotype‐specific canopy diameter across sites was substantially greater in 2014.

##### Height

Height showed a significant 3‐way interaction between S, E and T (*p* < .0001, Table S2). We conducted simple‐effects analyses for each year to explain the 3‐way interaction. Our main comparison is ecotypes within each site. Figure 3d shows estimated mean height (*±SE*) for ecotypes at each site in year 2010 and 2014 is presented in Figure S3. Overall, the general pattern of canopy height for both 2010 and 2014 appears consistent with CoGV (Figure 1e), with an ecotype‐specific increase in canopy height from west to east (Table S1). At the dry end (i.e., western KS and central KS), all ecotypes showed reduced height, with averages in the range of 14–37 cm high. However, for Western and central KS sites, the dry ecotype was significantly shorter than the mesic and wet ecotypes. No differences were detected between mesic and wet ecotypes. Moving east, ecotypes showed increasing canopy height, as much as 5.5 fold, and especially disproportionately for the much taller wet ecotype in the two wetter‐most sites (i.e., eastern KS and Illinois). A similar general pattern holds for 2014 (Table S1, Figure S3c), except the wet ecotype was substantially taller in 2014.

##### Blade width

We focus on significant main effects of ecotype (*p* = .0264) and site (*p* < .0001) on blade width, given the non‐significant interaction (*p* = .4162; Figures 1c, 3e, Table S2). Regardless of site, blade width differed among ecotypes such that wet ecotype leaves were wider (average 11.4 ± 0.38 mm) than mesic ecotype leaves (average 9.7 ± 0.38 mm) (Table S1). There was no evidence for differences in blade width between dry (average 10.7 ± 0.38 mm) compared to wet and mesic ecotypes at any of the sites. Considering site differences, leaf width was observed to increase (Figure 3e): western KS (8.0 ± 0.29 mm) < central KS (9.3 ± 0.28 mm) < eastern KS (11.2 ± 0.28 mm) < Illinois (14.0 ± 0.37 mm).

##### Differential canopy area response of ecotypes to rainfall gradient

**FIGURE 4:**
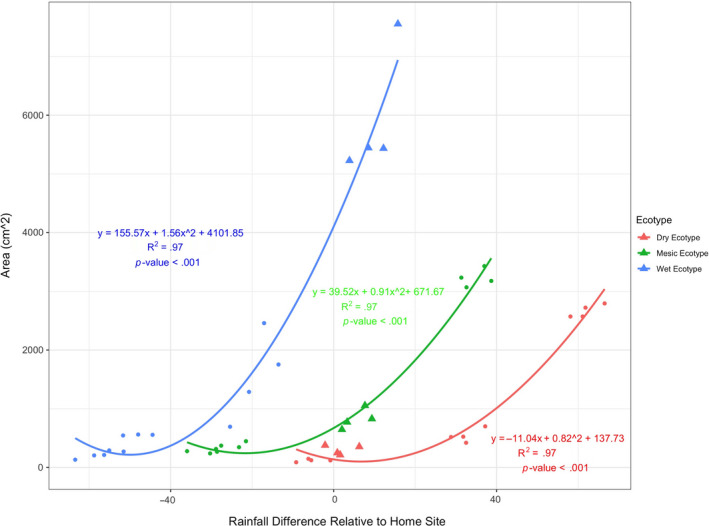
Fitted quadratic regression lines for canopy area (cm^2^) relative to difference in rainfall at the homesite for wet, mesic, and dry ecotypes, compared to rainfall at population source of origin. Note that the homesite is depicted by triangles

Canopy area, as scaled by difference in precipitation compared to homesite (Figure 4), showed that the wet ecotype canopy area was disproportionately more responsive to increased rainfall based on comparison of quadratic slopes of ecotype. (We used a quadratic function because this function fit better than a linear one.) The quadratic slope of wet ecotype response was estimated at 1.56 ± 0.22. That is, for every increase in one cm in precipitation, it corresponds to a 1.56 cm^2^ exponential increase in area (*p* < .001). The wet ecotype was more responsive to increased rainfall than the mesic ecotype (0.91 cm^2^ exponential increase in area (slope estimate 0.91 ± 0.11, *p* < .0001) and dry ecotype (0.82 cm^2^ exponential increase in area (slope estimate 0.82 ± 0.17, *p* < .0001). The quadratic slopes of mesic and dry ecotypes in response to rainfall differential were significantly different from the wet ecotype (both *p* < .0001) but dry and mesic ecotypes were not significantly different from each other. This indicates the wet ecotype is more responsive to precipitation.

#### 3.1.2 Biomass harvest

Vegetative and reproductive biomass both showed a significant 2‐way interaction between S and E (Table S2, both *p* < .0001). To explain these 2‐way interactions, we conduct simple‐effects analyses for both vegetative and reproductive biomass. Figure S4 shows estimated mean vegetative (A) and reproductive biomass (B) (±*SE*) for ecotypes at each site in 2011.

The general pattern of vegetative biomass appears consistent with CoGV (Figure 1e) and similar to the pattern for canopy area (Figure 3b), with an ecotype‐specific increase in vegetative biomass from west to east (Figure S4a). At the dry end of the gradient (i.e., western KS and central KS sites), all ecotypes showed small biomass and no evidence for differences between ecotypes (average 235.1 ± 58.28 g per plant). Moving east toward more favorable climates, the wet ecotype showed a disproportionately larger biomass relative to dry and mesic ecotypes in the two wetter‐most sites (i.e., eastern KS and Illinois), and especially in Illinois (1,124.9 ± 55.2 g per plant).

Moving on to reproductive biomass, the most striking pattern is that in Western KS, very little reproductive biomass was produced with no evidence for differences among ecotypes (mean 25 g per plant, Figure S4, Table S1). Moving eastward in central KS, reproductive biomass was significantly greater for the dry ecotype (103.0 ± 14.7 g per plant, Tables S1 and S2) while there was no evidence for differences between mesic and wet ecotypes (mean 46.3 ± 14.5 g per plant). From central KS to eastern KS, the pattern reverses and the wet ecotype is significantly greater (134.5 ± 14.5 g per plant) than dry and mesic ecotypes which are not different from each other (mean 72.9 ± 14.9 g per plant). At the wettest site, there is a disproportionate increase in reproductive biomass for the wet ecotype (307.7 ± 14.5 g per plant). In summary, the wet and dry ecotypes show evidence of local adaptation (Figures 1d and S4), but not the mesic ecotype, with highest biomass for the dry and wet ecotypes in their home environment.

#### 3.1.3 Reproductive responses

##### Probability of anthesis

**FIGURE 5:**
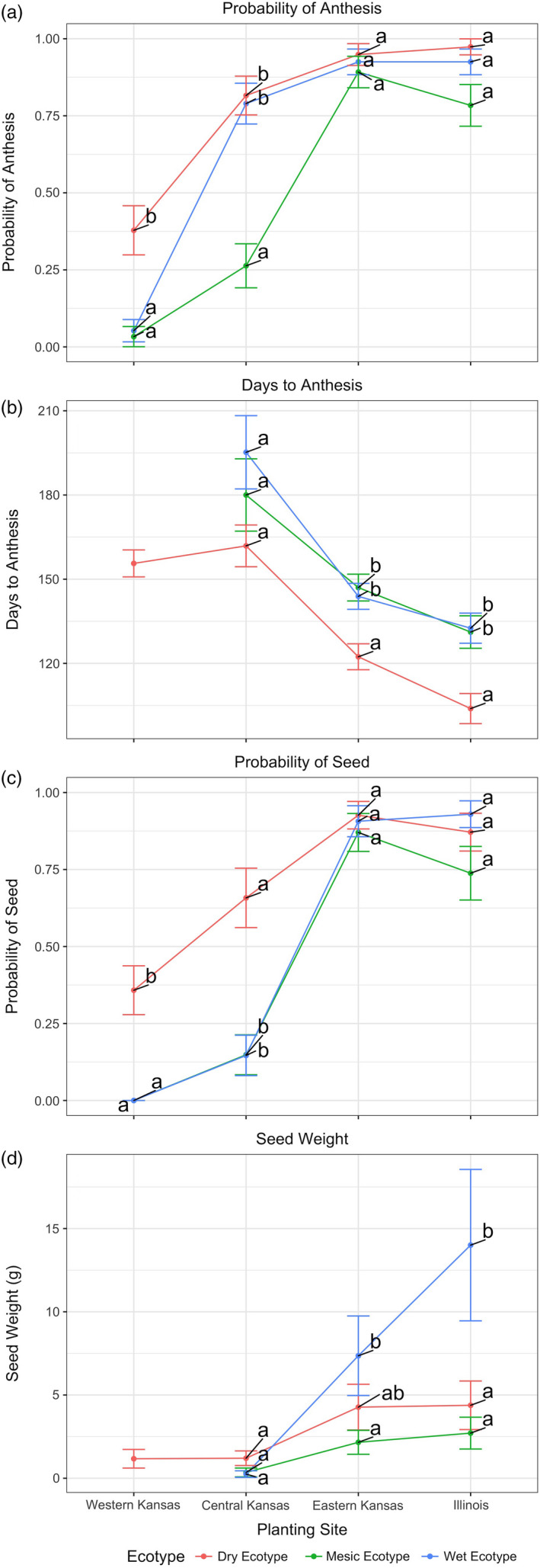
Least square mean estimates (±*SE*) of reproductive fitness traits for ecotypes (dry, mesic, wet) across reciprocal garden planting sites in Western KS (Colby KS), Central KS (Hays KS), Eastern KS (Manhattan KS), and Illinois (Carbondale Illinois). (a) Probability of anthesis, (b) days to anthesis, (c) probability of seed production, (d) seed mass. Sites with different letters indicate significant differences within a site

Main effects of ecotype (*p* = .004) and site (*p* < .0001) were significant for the probability of anthesis (Figures 1c, 5a, Tables S1 and S2) with no evidence for interaction (*p* = .1649). At the driest site, in western KS, probability of reaching anthesis was drastically reduced and close to 0 for both wet and mesic ecotypes (probability .053 ± 0.07 and 0.033 ± 0.03, respectively) compared to the dry ecotype (0.38 ± 0.08). Moving eastward to central KS, the wet (0.79 ± 0.07) and dry ecotypes (0.82 ± 0.06) were significantly more likely to reach anthesis than the mesic ecotype. In eastern KS and Illinois, probability of anthesis was maximized for all ecotypes (wet 0.93 ± 0.04, dry ecotype 0.95 ± 0.04, and mesic 0.89 ± 0.05). For all ecotypes, plants were significantly more likely to reach anthesis on the easternmost sites relative to westernmost planting sites.

##### Days to anthesis

For those plants that did reach anthesis, days to anthesis differed by ecotype (*p* = .0019) and site (*p* < .0001), but no evidence for any interaction was apparent (*p* = .7543; Table S2, Figures 1c, 5b). Within a site, the dry ecotype flowered sooner than other ecotypes, with some site‐specific differences. In western KS, the dry ecotype was the only ecotype to flower (by October 13) and reached anthesis at approximately 157 ± 5 Julian days. In central KS, there was no evidence for differences in days to anthesis among ecotypes (Table S2; wet 195 ± 13 days, mesic 180 ± 13 days, dry 162 ± 7 days). In eastern KS and Illinois sites, the dry ecotype flowered significantly earlier than the mesic and wet ecotypes, but the latter two showed no evidence of differences in days to anthesis. In summary, the dry ecotype flowered sooner than wet and mesic ecotypes by about 20 days (Table S1, Figure 5). In comparing sites, all ecotypes flowered earlier going eastward by about 60 days (180 Julian days in western KS versus 120 days in Illinois; Table S1, Figure 5).

##### Probability of producing seed

The site main effect (*p* < .0001) was confounded with a S × E interaction (*p* = .0330; Table S2) so we conducted simple‐effects analyses with main comparison being ecotypes within each site. (Figure 5c, Table S1). The most striking pattern was observed in western KS, whereby the probability of producing seed was estimated at 0.36 ± 0.08 for the dry ecotype, but was negligible for other ecotypes, primarily because they never reached anthesis (Figure 5b). In central KS, probability of seed production was not significantly different (Table S2) between wet and mesic (0.15 ± 0.07) ecotypes, but probability was significantly less than dry ecotype in Central KS (0.66 ± 0.10). Going from central KS to eastern KS, probability of producing seed increased for all ecotypes (wet 0.91 ± 0.05, mesic 0.87 ± 0.06, dry 0.93 ± 0.05) but was not significantly different among ecotypes in Eastern KS. Going to the wettest site, there is no significant difference in probability compared to eastern KS and no significant difference among ecotypes there (wet 0.93 ± 0.04, mesic 0.74 ± 0.09, dry 0.87 ± 0.06), but the probability was high as conditions become mesic and wet.

##### Seed weight

Because site (S) main effect (*p* < .0001) was confounded with a S × E interaction (*p* = .0081; Table S2), we conducted simple‐effects analyses with the main comparison being ecotypes within each site (Figures 1e, 5d and Table S1, CoGV). The most striking pattern is that in the western KS site, the dry ecotype was the only ecotype producing seed (1.17 ± 0.56 g), albeit a small amount, while the other ecotypes failed entirely to produce seed because they did not flower there. Moving eastward in central KS, seed weights were similarly as low and were not significantly different among ecotypes (Table S1; wet 0.25 ± 0.20 g, mesic 0.35 ± 0.26 g, and dry 1.20 ± 0.44 g). From Central KS to Eastern KS sites, seed weight increased for all ecotypes (wet 7.36 ± 2.39 g, mesic 2.16 ± 0.72 g, dry 4.27 ± 1.37 g) but only mesic differed from wet ecotype. Finally, in the wettest site, there is no significant increase in seed weight compared to eastern KS site. However, the wet ecotype (14.01 ± 4.55 g) was significantly greater than dry (4.38 ± 1.46 g) and mesic ecotypes (2.71 ± 0.97 g), with no evidence for differences between dry and mesic ecotypes. Much of the pattern detected for seed weight mirrors that of reproductive biomass such that seed mass increased west to east. However, with reproductive biomass we see evidence of local adaptation (Figure 1d) of the dry and mesic ecotypes, not seen in seed weight in 2012.

### 3.2 Homesite comparisons shows suite of ecotype‐specific traits and climate controls

#### 3.2.1 Subset of homesite response variables

For this dataset, we consider response variables measured on ecotypes in their homesite (dry ecotype in central KS, mesic ecotype in eastern KS, and wet ecotype in Illinois). Starting with vegetative variables, for emergence, there was no evidence for difference in ecotype emergence (Figure S5a) in dry and mesic homesites, but ecotype emergence in dry and mesic homesites were slightly but significantly later (*p* < .0001) than the wet ecotype, which emerged 4 days sooner in its homesite. For the remaining vegetative responses (Figure S5b‐f), all showed increases going west to east as climate gets more favorable (central KS < eastern KS < Illinois; Table S1). Canopy area for dry ecotype (236 ± 44 cm^2^) was significantly smaller (38%, *p* = .0007) than mesic ecotype canopy area (615 ± 118 cm^2^), which was significantly smaller (11%, *p* < .0001) than wet ecotype canopy area (5,479 ± 1,041 cm^2^). Also, dry ecotype diameter in central KS (36 ± 3 cm) was significantly smaller (*p* < .0001) than mesic ecotype diameter in eastern KS (60 ± 4 cm) which in turn was significantly smaller (*p* < .0001) than wet ecotype diameter in Illinois (127 ± 4 cm). For height, dry ecotype (23 ± 1 cm) was significantly shorter (*p* < .0001) than the mesic ecotype (48 ± 3 cm) and significantly shorter (*p* < .0001) than wet ecotype (110 ± 6 cm). For blade width, dry ecotype (9.49 ± 0.46 mm) was not significantly different than mesic ecotype blade width (10.40 ± 0.46 mm) but significantly narrower (*p* < .0001) than blade width of wet ecotype (15.58 ± 0.62 mm). For both vegetative and reproductive biomass, there was no evidence of differences between the dry and mesic ecotypes and both were significantly smaller than the wet ecotype.

Looking at reproductive variables, for probability of anthesis, there were no significant differences among ecotypes in their respective homesite (dry ecotype 0.82 ± 0.06, mesic: 0.89 ± 0.05; wet: 0.93 ± 0.04). For the remaining variables, they generally varied from west to east (Figure S5g–k, Table S1). Days to anthesis was longer for the dry ecotype (estimated 162 ± 7 days) compared to the wet ecotype (132.5 ± 5 days). However, the mesic ecotype (147 ± 5 days) showed intermediate days to anthesis and no evidence for difference from the dry or wet ecotype in homesites (Table S1). The dry ecotype had a 0.66 ± 0.09 probability of seed production but was significantly less (*p* = .022) than probability of seed production for the wet ecotype (0.93 ± 0.04). The mesic ecotype was intermediate (0.87 ± 0.06) and showed no evidence for differences from wet and dry ecotypes in their homesites. Fitness in terms of grams of seed per plant was greatest for the wet ecotype (14.01 ± 4.55 g) relative to the mesic ecotype (2.16g ± 0.72 g) and dry ecotype (1.20 ± 0.44 g).

#### 3.2.2 Random forest modeling of ecotype traits in homesite

**FIGURE 6:**
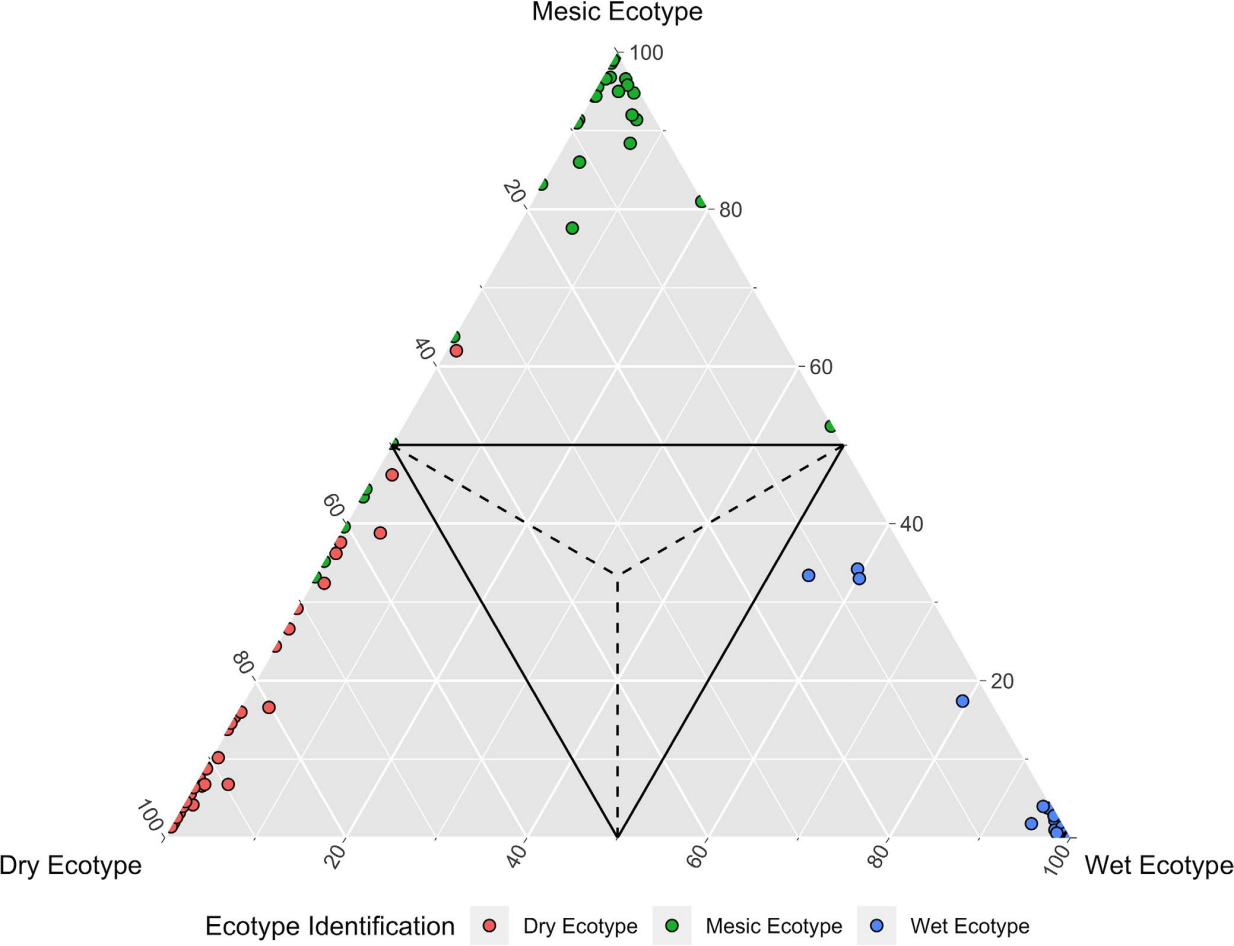
Classification plot obtained from the random forest analyses showing training/validation triangle with percent votes of the individuals from the 10 fold‐cross validation from random forest training and validation set. Each point is an individual. Dry ecotype is denoted in red, mesic ecotype is denoted in green, and wet ecotype is denoted in blue. Individuals within the solid lines indicate individuals with poor discernment of the algorithm (<50% votes). Plants falling outside of the solid lines are clearly discerned; that is, more than 50% of votes from the validation were for that ecotype

The random forest classification approach corroborated the traits of ecotype morphologies and reproductive fitness in the homesite comparison. We assigned to one of three ecotypes (dry, mesic, wet) with accuracy of 94.3% (Table S3, Figure 6). Highest rate of misclassification occurred with individuals of the mesic ecotype, with 4.7% (6 plants) incorrectly classified as dry ecotype and 1% (1 plant) of dry ecotype incorrectly classified as mesic ecotype. Importantly, the wet ecotype in its home environment was never misclassified (Table S3). The classification performance of random forests appears to support distinct vegetative and reproductive morphologies, consistent with three ecotypes.

#### 3.2.3 PCA of traits from ecotypes grown in their homesites and associations with climate variables from population source of origin

**FIGURE 7:**
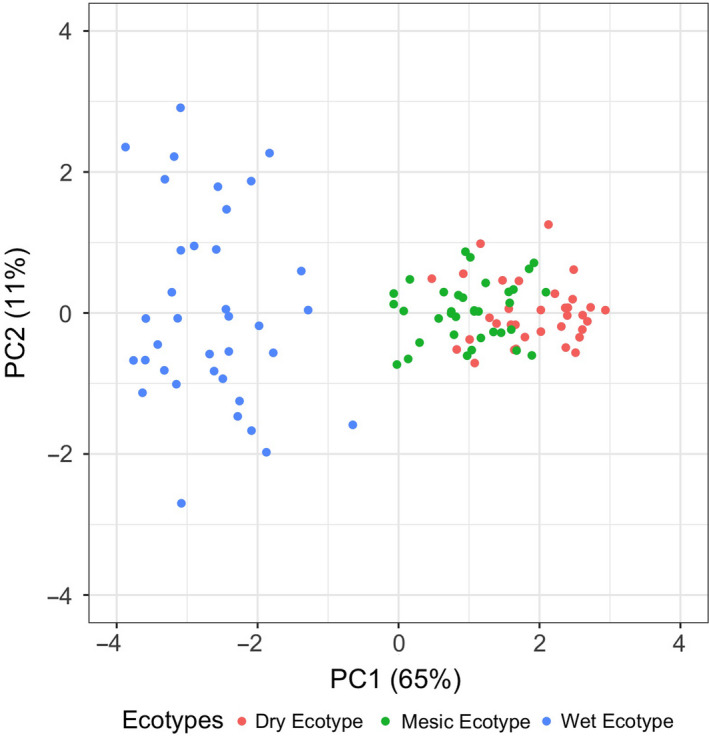
Scatter plot of the first two principal component scores for vegetative and reproductive traits corresponding to 106 plants corresponding to ecotypes growing in their homesite. Abbreviations and symbols correspond to regional ecotypes (Red = dry ecotype from Central Kansas; Green = mesic ecotype from Eastern Kansas; Blue = wet ecotype from Illinois). Mesic and dry ecotypes in their home environments were differentiated from the wet ecotype in Illinois prairies mostly along the first PCA axis

Using principal components analysis to also characterize the ecotypes, we identified ecotype‐specific traits that characterized each ecotype in its home environment. Traits for the wet ecotype represent an especially distinct assemblage compared to dry and mesic ecotypes in their home environments. Scatter plot of PC1 and PC2 of vegetative and reproductive response variables (Figure 7, Table S3) show main clustering of the wet ecotype with overlap between the dry and mesic ecotypes. Scree plots indicate over 60% of variation is explained by PC1 (Figure 7), whereas the first three PC axes account for 80% of the variation in the data. PC1 seemed to be heavily influenced by all variables, as all loadings had an absolute value greater than 0.50. Specifically, in order of loadings, diameter (loading −0.95), height (loading −0.94), canopy area (loading −0.93), vegetative emergence (loading 0.83), blade width (loading −0.76), seed weight (loading −0.57), and, finally, days to anthesis (loading 0.55).

Next, we used stepwise regression to evaluate association between ecotypes from PCA scores to home‐climate variables to determine which climate variables are important in explaining ecotypic variation. Scores for PC1 (Table S4) were negatively associated with seasonal diurnal mean temperature variation (−0.65 ± 0.21) and number of precipitation events (−0.255 ± 0.022) and positively associated with seasonal mean precipitation (0.08 ± 0.02), seasonal mean temperature (0.40 ± 0.09), and annual diurnal temperature variation (1.61 ± 0.20). Scores corresponding to PC2 and PC3 were not significantly associated with any climatic variables (results not shown).

### 3.3 Genetic variation among ecotypes aligns with phenotypic variation

#### 3.3.1 Divergence and diversity

**FIGURE 8:**
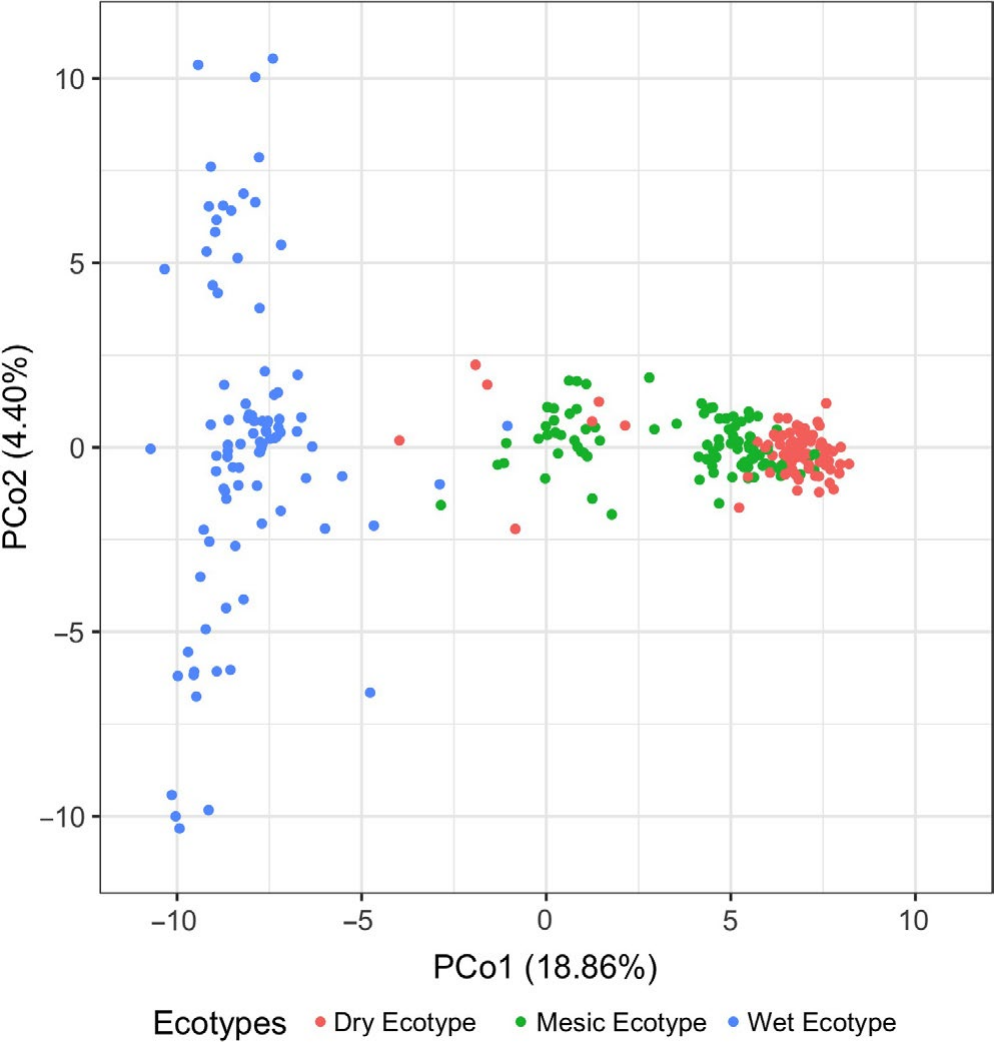
Scatter plot of the first two principal coordinates scores for allelic frequency of 4,641 SNP marker loci. There were 314 plants genotyped (110 dry, 106 mesic, and 98 wet ecotype). Abbreviations and symbols correspond to regional ecotypes (Red = dry ecotype from Central Kansas; Green = mesic ecotype from Eastern Kansas; Blue = wet ecotype from Illinois). Mesic and dry ecotypes in their home environments were differentiated from the wet ecotype in Illinois prairies mostly along the first axis

Mesic and dry ecotypes are genetically differentiated from wet ecotype, with reduced diversity in wet ecotype. Principal coordinate analysis (PCoA) of SNP allelic frequencies revealed sorting of wet ecotype as a distinct cluster with partial overlap between dry and mesic ecotype individuals (Figure 8) as was the case for PCA of morphological phenotypes (Figure 7). Scree plots indicate that 18.8% of variation was explained by PCo 1, whereas 4.4% and 4.1% were explained by PCo 2 and 3, respectively. In assessing the association between PCo scores 1 and 2 to environmental conditions, the stepwise selection approach showed that mean annual precipitation (−0.38 ± 0.02) and seasonal precipitation (0.50 ± 0.06) were significantly associated to PCo 1 (Table S5) and annual mean temperature (−1.54 ± 0.04), mean annual precipitation (−0.23 ± 0.02), number of precipitation events <1.25 cm (1.03 ± 0.10), and seasonal mean temperature (1.51 ± 0.24) with PCo 2.

The extent of pairwise genetic differentiation among populations is characterized by *F*_st_ statistics (Table S6). As expected, populations within a given ecotype show low pairwise genetic distances. Comparing between ecotypes, pairwise *F*_st_ values (1) between populations of mesic and dry ecotypes *F*_st_ average is 0.013, (2) between populations of wet and mesic ecotypes *F*_st_ average is 0.028, and (3) between the wet and dry ecotypes *F*_ST_ average is 0.030. (*F*_sr_ estimates: Dry ecotype=0.012, mesic ecotype=0.013, wet ecotype=0.021) The greatest genetic distance was apparent between populations from the wet ecotype relative to populations from mesic or dry ecotypes with *F*_st_ as high as 0.037. Pairs of populations with *F*_st_ values of 0.028 or below are considered to have undergone slight neutral differentiation (Meirmans & Hedrick, 2011).

#### 3.3.2 Genetic outlier analyses and associations with climate

**FIGURE 9:**
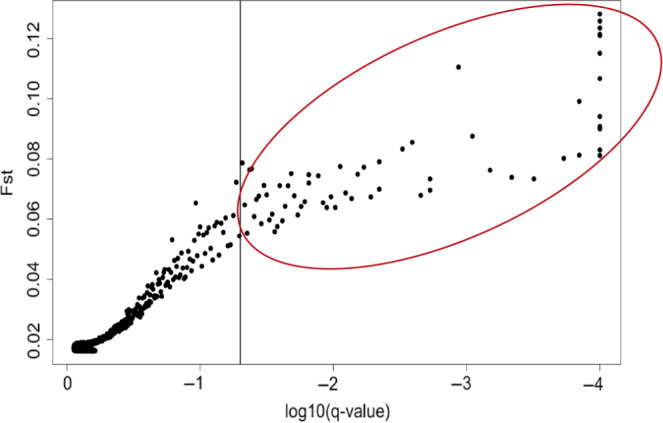
Scatter plot of Fst values as a function of statistical significance of SNP markers, as obtained using *Bayescan v2.1.* Points to the right of the vertical line indicates 64 markers with significant evidence of divergent selection among populations based on a q‐value (i.e., *p*‐value adjusted for FDR) lower than .05

We identified 64 SNPs in *BayeScan* showing significant divergent selection across populations (Figure 9), 18 of which were annotated (See Table S6). We also used *Bayen*v2 to identify outliers and provided a list of consensus outliers between methods (Table S7). Using two separate approaches allows us to obtain consensus outlier loci to strengthen inferences on selection. For outlier analysis using *Bayenv2*, the top 1% of the X^T^X values comprised 46 SNPs, about half of which had annotations. Candidate gene functions included NAC transcription factors, peroxidases, glutamate synthase, and GA1 (Sb01g021990.1) (Table S7), among others. One of the SNP outliers found within a gene of interest and identified in both *BayeScan v2.1* and *Bayenv2* was GA1, which ranked as 14th highest X^T^X differentiated SNP. Gene GA1 codes for a gene ent‐copalyl diphosphate synthase that is involved with the first step in the synthesis of gibberellic acid (Hedden & Thomas, 2012).

We used *Bayescenv* to assess association between SNP allelic frequency to environmental conditions; out of total 4,641 SNP considered, a subset of 440 SNPs showed significant associations (*q*‐value < 0 0.05) with at least one environmental factor (Figure S5, Table S8). (Note that SNPs were often associated with more than one environmental factor). Of those 440 SNPs, the greatest number showed a significant association (*q*‐value < 0.05) with seasonal rainfall (96 SNPs) and secondarily, with aspects of temperature (mean annual temperature 76 SNPs, seasonal diurnal variation 60 SNPs). Second, we took only those SNPs identified as outliers in *Bayescan* and *Bayenv* (110, Table S7) and associated their occurrence to climate (Figure S5). Of the SNPs identified as outliers from *Bayescan and Bayenv*, the greatest number of significant associations were significantly associated (*q*‐value < 0.05) with seasonal mean precipitation (41 SNPs), seasonal diurnal variation (29 SNPs), and seasonal annual temperature (25 SNPs) (Table S8, Figure S5).

We used pRDA analyses of genetic variation to quantify relative importance of climate versus geography in the full model (model 1) that incorporates both climate and geography (Table S9). In the second model in which geography explained genetic variation conditioned on climate, total variance explained was 15%. In the third model in which climate variables explained genetic variation conditioned on geography, total variance explained was 74%. Thus, climate structured genetic diversity more than geography (latitude and longitude). Total joint explained varians was 89%, leaving 11% unexplained by joint geography and climate variables. Geographic and environmental loadings of the full model (Table S10) showed that precipitation variables dominated loadings on pRDA1 and temperature variables explained loadings on pRDA2.

#### 3.3.3 GWAS: Relating genotype to phenotype

Using *TASSEL* to associate genotype and phenotype, 163 SNPs were significantly associated to a morphological variable. Number of SNP associations were height 87, emergence 38, canopy area 28, blade width 7, diameter 2, and anthesis 1 (Table S11). Of the 163 significant SNPs, 33 were also identified as outliers (Table S7), with canopy area having 9 SNP associations, height 11, anthesis 1, and emergence 12. Several of these SNP stand out as being functionally significant based on annotations, notably HB‐3 transcription factor, GA‐1 ent‐copalyl diphosphate synthase/ magnesium ion binding, BAN (BANYULS), oxidoreductase, and WOX11 (WUSCHEL related homeobox 11) DNA binding/ transcription factor (Table S11).

**FIGURE 10:**
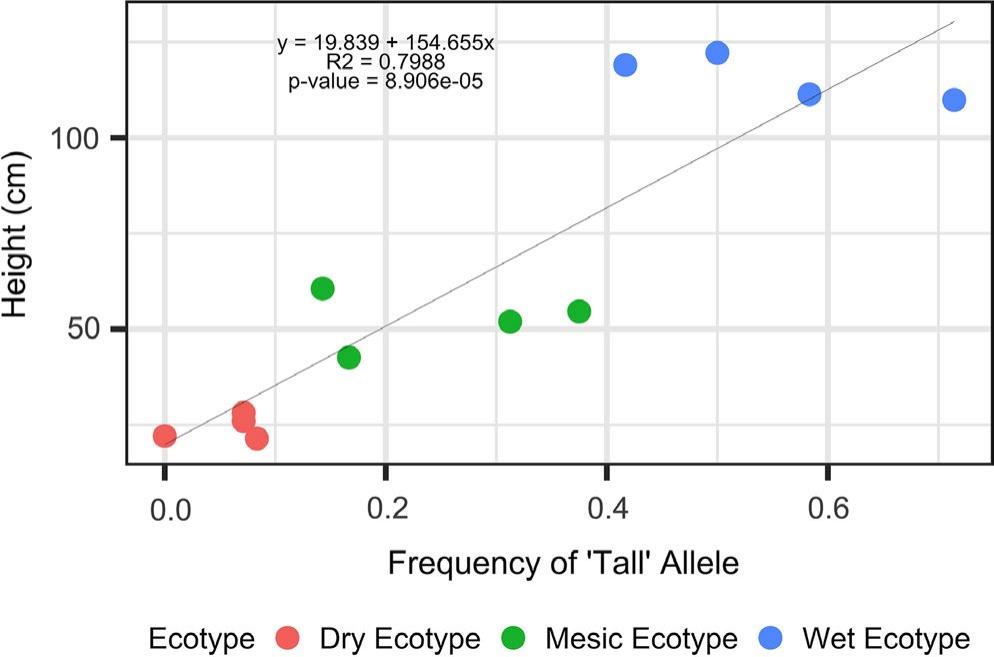
Scatter plot and fitted regression line depicting average population plant height as a function of allelic frequency for the “tall” allele of the GA1 outlier. Each population is color‐coded by ecotype. Red = dry ecotype, green = mesic ecotype, blue = wet ecotype. The four points per ecotype represent the four source populations

Most notably, the SNP within the GA1 gene showed association with height in ecotypes (GWAS) and was also a genetic outlier in both *Bayescan* and *Bayenv2* (Table S7). We regressed plant height as a function of GA1 frequency and showed evidence for a linear relationship GA 1 SNP variation and height. Results showed the “tall allele” (arbitrarily, the allele that dominates in tall plants; Figure 10) had the greatest frequency in the wet ecotype, intermediate frequency in mesic ecotype, and lowest frequency in dry ecotype, each in their home environments. The wet ecotype was about 5x taller than dry ecotype in their homesites (Figures S1 and S5).

## 4 DISCUSSION

Here, we document ecotypic variation in the dominant prairie grass *A. gerardii* across the spatially varying climatic gradient of the US Great Plains. We found evidence for local adaptation only for reproductive biomass in wet and dry ecotypes. We observed strong CoGV in most vegetative traits (Conover et al., 2009; Conover & Schultz, 1995; Crispo, 2008). Further, homesite comparisons clearly highlight the extent to which ecotypes have adaptively diverged in their homesite. Finally, phenotypic differences among ecotypes are underpinned by genetic differences and outliers in traits such as height and stress response. We found a notable outlier SNP in GA1 whose allele frequency varies clinically with height of ecotypes across the Great Plains. Genetic outliers show climate associations, primarily with growing season precipitation and secondarily growing season temperature. Below we delve into reciprocal garden phenotypic responses, homesite trait syndromes, genetic bases for traits, and finally implications for climate change and restoration.

### 4.1 Phenotypic variation in reciprocal gardens show role for plasticity and genetics

Transplant experiments are ideal to investigate forms of phenotypic variation and to quantify the extent to which phenotypic differences across environmental gradients are caused by genetic variation and/or phenotypic plasticity (Clausen et al., 1940; McMillan, 1959, 1965). A number of studies have investigated sources of variation of plant performance in environments with contrasting climate selection pressures (Clausen et al., 1940; Schmid, 1985; Weber & Schmid, 1998; Etterson, 2004), all using reciprocal transplant experiments. Both plasticity and genetic variation have been widely observed in many settings, such as altitude (Byars et al., 2007; Clausen et al., 1940; Gonzalo‐Turpin & Hazard, 2009), precipitation gradients (Anderson et al., 2015; Eckhart et al., 2004), and latitudinal clines (Chapin & Chapin, 1981; McGraw et al., 2015; McMillan, 1959, 1965).

We expected a balance between environmental phenotypic plasticity versus genetic adaptive variation (Bradshaw, 1984; Linhart & Grant, 1996) due to differing strength of spatially varying selective forces such as precipitation. If selection from long‐term climate were strong, especially due to strong spatial heterogeneity in rainfall (Axelrod, 1985), local adaptation to climate could be expected (Galliart et al., 2019). Local adaptation is defined as an interaction between ecotype and site, with ecotypes from local populations outperforming non‐local transplants in different climates (Linhart & Grant, 1996) and depicted as S × E (Figure 1). Interestingly, local adaptation was only observed in for reproductive biomass (Figure S4b) in wet and dry ecotypes. However, we did observe complicated patterns of S, E, and S × E interactions. Next, we consider the types and strength of these effects and their patterns of interaction.

We detected environmentally relevant S × E interactions, especially for seed production (Figure 5d). For example, only the dry ecotype produced seed in Western KS. Conversely, all ecotypes produced seed on the wet end of the gradient, though the wet ecotype produced significantly more seed compared to dry and mesic ecotypes under the favorable conditions of Illinois. This suggests that spatially varying climate may be imparting strong selection on reproductive fitness in these ecotypes and causing differentiation for this key fitness trait.

Ecotypes also differed in terms of flowering time. The dry ecotype flowered earlier (21–30 days) compared to the other ecotypes depending on site. This is a putative adaptation to speed up reproduction in response to end of season drought in central and western KS. Early flowering time in response to drought has also been observed in reciprocal garden studies of *Clarkia* (Eckhart et al., 2004) and in experimental evolution studies with *Mimulus* (Dickman, Pennington, Franks, & Sexton, 2019) and *Brassica* (Hamann, Weis, & Franks, 2018). However, most examples of changes in flowering time deal with latitude and day length (McMillan, 1959, 1965) or coastal‐inland gradients (Lowry et al., 2009). Strong co‐gradient plasticity of phenology across environmental gradients seen in *Rhinanthus minor* could provide a mechanism to buffer against variable climates (Ensing & Eckert, 2019). In contrast, the flowering time differences observed here portends for beginnings of genetically based reproductive isolation (Nosil, 2012) especially between wet and dry ecotypes, further reductions in gene flow, and ultimately speciation.

In contrast with reproductive traits, most vegetative response variables showed a pattern of CoGV (Figure 1e). CoGV was observed such that at the dry end of the gradient in western KS site, several vegetative responses (canopy area, height, diameter) were similar in magnitude and did not show evidence for differences among ecotypes there. Presumably, harsh dry conditions minimized ecotype trait differences, even for the local ecotype. Small stature is expected to be favored as an adaptation to reduce water loss in dry and windy conditions of the western KS reciprocal gardens (Kramer et al., 2018) and in other studies (Byars et al., 2007; Eckhart et al., 2004). Moving eastward into more favorable, mesic environments, ecotype differences in vegetative traits became more apparent and showed clear signs of CoGV (Figures 1e, 3b–d, and S4). Nearly all vegetative phenotypes of the three ecotypes increased in magnitude under more favorable conditions of increasing rainfall, with the wet ecotype responding disproportionately greater than the other two ecotypes across the gradient (Figures 3b–d, 4, Figure S4). Our results agree with a recent review (Conover et al., 2009) that documented that CoGV was often associated with morphological variables. This is a similar pattern observed along altitudinal clines (Clausen et al., 1940), such as *Potentilla glandulosa* in the Sierra Nevada Mountains, *Poa* in Australian mountain (Byars et al., 2007), and *Festuca* in the Pyrenees (Gonzalo‐Turpin & Hazard, 2009). In stressful environments, such as high elevations or the dry, water‐limited western Great Plains, plants putatively allocate more resources to growth and survival than flowering and seed production (Bloom, Chapin, & Mooney, 1985; Chapin, 1991; Gonzalo‐Turpin & Hazard, 2009; Harper, 1977; Harper & Ogden, 1970). In more favorable habitats, plants produce more seed. In the moister, favorable environment of Illinois, ecotypes showed greatly increased seed production, especially of the local ecotype.

Interestingly, our results do not agree with recent studies showing local adaptation of vegetative cover of wet and dry ecotypes in seeded community plots over 5 years (Galliart et al., 2019) where ecotypes grew with other species, as they would in nature. In the community plots, ecotypes compete with themselves and other species for resources such as light, nutrients, and water. In contrast, the single plants of this reciprocal garden experiment grew without competition and rarely showed signs of local adaptation. Apparently, only in a realistic community do we observe local adaptation expressed in these ecotypes, showing the importance of biotic interactions in driving local adaptation (Bischoff et al., 2006; Grassein, Lavorel, & Till‐Bottraud, 2014; Galliart et al., 2019), thus highlighting the need to put local adaptation in the context of the community.

### 4.2 Homesite comparisons show suites of traits that distinguish ecotypes

Homesite comparisons highlight the extent to which these ecotypes have adaptively diverged. Homesite comparisons are also aligned with random forest‐based classification and PCA scatter plots, both showing distinctive trait assemblages for each ecotype. Traits often respond to selection by climate (Chapin, 1991; Chapin, Autumn, & Pugnaire, 1993) and are often correlated, resulting in adaptive trait syndromes (Ackerly et al., 2000; Aspinwall et al., 2013). Our results also agree with recent meta‐analyses of plant form in grasses such that grasses of the wetter, limited environments were characterized by broad leaves (Gallaher et al., 2019). In contrast, grasses of open habitats were characterized by narrow, short leaves (Gallaher et al., 2019), putatively associated with water‐limited open, arid environments.

In terms of adaptive trait syndromes, in the wettest site, plants of the wet ecotype were robust, tall, with large canopy, wide diameter, high vegetative biomass, and broad leaves. These traits may allow the wet ecotype to compete with the abundant tall forbs and shrubs in the highly competitive, species‐dense, light‐limited prairie peninsula of Illinois (Kuchler, 1964). Indeed, height is a major determinant of a plant's ability to compete for light (Moles et al., 2009), based on global analyses. The wet ecotype in its climate characterized by adequate moisture and temperature also had a high probability of flowering and producing large quantities of seed. Similarly, at Konza Prairie, *A. gerardii* produced more flowering stalks in wetter years (La Pierre et al., 2011) and showed reduced flowering stalk production under rainout shelters (Swemmer, Knapp, & Smith, 2006). Similar to results from Eckhart et al. (2004), in our study, the wet ecotype took the shortest time to flower in its home environment (132.5 days) because it putatively experiences greater number of growing degree days sooner in the moderate climate of Illinois compared to other sites (3,799 growing degree days in Hays, KS versus 4,087 in Illinois; Johnson et al., 2015).

On the other hand, at the dry end of the gradient, plants were dwarfed, with small diameter, reduced canopy area (Kramer et al., 2018) and low vegetative biomass and narrow leaves, indicative of a water‐limited environment. Yet, the dry ecotype flowered soonest out of the ecotypes, perhaps due to harsher conditions (Table 1), and shorter growing degree days there. Thus, growth form and fitness were limited by the harsh water‐limited environment of central KS. Put in broader terms, the local ecotype must adjust growth to match its limited resources (Chapin, 1991; Chapin et al., 1993), putatively water in central KS and light in Illinois.

Extensive phenotypic variation of *A. gerardii* has been observed from this experimental reciprocal garden platform in anatomy (Olsen et al., 2013), chlorophyll absorbance (Caudle et al., 2014), root production (Mendola et al. 2015), photosynthesis (Maricle et al., 2017), stomates (Varvel et al., 2018), and morphology (Kramer et al., 2018). In terms of plant productivity across the Great Plains climate gradient, Epstein, Lauenroth, Burke, and Coffin (1996) reported an increase in productivity with increased rainfall along a longitudinal gradient for *A. gerardii*. Avolio and Smith (2013) also found phenotypic variation in *A. gerardii* in experimental rainfall manipulations. Looking at temperature, in a study of switchgrass genotypes from varying latitudes of origin, Aspinwall et al. (2013) found evidence for the role of temperature in a suite of adaptive traits related to morphology and physiology.

### 4.3 Genetic differences underlying phenotypes

Phenotypes were structured, in part, by genetic differences among ecotypes (Gray et al.., 2014; Price, Salon, & Casler, 2012). The wet ecotype was sharply differentiated from the dry ecotype in terms of morphology and reproductive features. In spite of having low *F*_st_ and low levels of neutral genetic differentiation among populations and ecotypes, presumably selection pressures associated with mainly precipitation were strong enough to maintain differences in spite of gene flow. Other studies have found differentiation in spite of gene flow (Gonzalo‐Turpin & Hazard, 2009). Importantly, the strong differences in flowering time between wet and dry ecotypes portend the future reduction of gene flow and reproductive isolation.

Genetic outliers were likely of adaptive significance and suggest divergent selection, presumably due to selection from spatially varying climate. Ecotypes differed in terms of candidate genes such as NAC transcription factor, glutamate synthase, peroxidase, and GA1. The SNP in GA1 had an allele frequency that varied clinically across the Great Plains and has high ecological and functional significance (Figure 10) with wet ecotypes growing 4.7 times taller than the dry ecotype, Figures S1 and S5). Expression of GA1 controls internode length and consequently height (Milach, Rines, & Phillips, 2002). Height correlates with increased biomass, and putatively greater competitiveness (Moles et al., 2009), as would be advantageous in wet, light‐limited prairie peninsula dominated by tall forbs and shrubs (Kuchler, 1964). Conversely, the dry ecotype would be advantaged by short stature to reduce evaporative loss as an adaptation to water‐limited climates such as central and western KS (Kramer et al., 2018; Maricle et al., 2017). Other studies have also identified other candidate genes across altitudinal gradients (Pluess et al., 2016; Rellstab et al., 2016), latitudinal gradients (Hancock et al., 2011), and precipitation gradients (Exposito‐Alonso et al., 2017). These studies provide powerful insight into candidate genes and genetic mechanisms responsible for adaptive divergence.

Outlier SNPs identified in *Bayscanenv* showed a clear relationship with climate especially precipitation and temperature variables. Furthermore, pRDA shows that climate, more than geography structures genetic variation. Our study took an approach using outlier candidate genes across gradients, that is, genome–environment associations as highlighted in recent reviews (Bragg, Supple, Andrew, & Borevitz, 2015; Laskey, Forester, & Reimherr, 2018; Rellstab, Gugerli, Eckert, Hancock, & Holdregger, 2015; Sork, 2016).

Recent empirical studies have detected genome–environment associations. *Arabidopsis halleri* showed genomic footprints of selection to altitude in the Alps (Fischer et al., 2013). Laskey et al. (2012) used redundancy analyses to quantify the association between climate, geography, and genomics in Eurasian *Arabidopsis* populations and discovered early spring temperature explained most of the variation. Pluess et al. (2016) related phenology candidate genes to climate, geographic, and seasonality in European beeches. Finally, Exposito‐Alonso et al. (2017) linked genetic variation to drought tolerance in *Arabidopsis* accessions from contrasting climates and highlighted the role of within‐species variation in the evolutionary response to climate.

### 4.4 Implications for climate change

Understanding how climate structures genetics, form, and function of a dominant grass species is critical to predicting grassland response to climate change. Several lines of evidence suggest that climate, especially seasonal precipitation and temperature variation, structures *A. gerardii* ecotype form and genetic variation. Other studies of *A. gerardii* using redundancy analyses corroborate that climate, more than geographic location (distance), structures neutral genetic variation (Galliart et al., 2019). Third, genetic outliers based on *Bayscanenv* were related to both seasonal precipitation and temperature variation. Precipitation and temperature patterns have been in place for the last 10,000 years (Axelrod, 1985), leading to selective pressure and ultimately adaptive differentiation. Our study showed a complex mosaic of abiotic stressors, not just precipitation, across the Great Plains to which *A. gerardii* must adapt either genetically and/or plastically. Adaptation was observed with experimental manipulation of rainfall and temperature (Ravenscroft, Whitlock, & Fridley, 2015), showing some genotypes were preferred in different experimental treatments. Resurrection experiments show selection after drought (Dickman et al., 2019; Hamann et al., 2018). *Arabidopsis* ecotypes tolerated extreme drought (Exposito‐Alonso et al., 2017) through within‐species variation in drought tolerance and its evolutionary response to climate.

Such knowledge of intraspecific variation across precipitation gradients and the relative importance of plasticity versus genetic adaptive variation are critical to predict and model grassland biome responses to climate change (Aspinwall et al., 2013; Smith, Alsdurf, Knapp, Baer, & Johnson, 2017; Yurkonis & Harris, 2019). This knowledge is urgently needed to predict grassland response to warmer and drier summers in the Great Plains. The year 2012 was the worst drought in 50 years with unprecedented “mega‐droughts” predicted for the central US (Cook et al., 2015). A recent phenotypic modeling study predicted that by 2070, with climate change, populations of short‐statured, dwarf forms of *A. gerardii* from dry parts of its range would be favored ~800 km eastward and result in 60% decrease in biomass, if it can migrate there in time or be planted through restoration (Smith et al., 2017). Reduction in productivity could have cascading effects on prairie function (Hoover, Knapp, & Smith, 2014; Knapp et al., 1998), cattle forage production (Gibson, Espeland, et al., 2016; Gibson, Donatelli, et al., 2016), grassland restoration (Baer, Gibson, & Johnson, 2019), and conservation.

### 4.5 Informing restoration

Tallgrass prairie, one of the most diverse grasslands, is critically endangered (Hoekstra et al., 2005) with only 4% native prairie remaining (Samson & Knopf, 1994). For example, less than 1,000 ha remain in eastern tallgrass prairie compared to the original 9 million ha (Samson & Knopf, 1994). Our study informs land management and restoration strategies because knowledge of climate‐matched ecotypes (Hufford & Mazer, 2003; McKay, Christian, Harrison, & Rice, 2005; Nicotra et al., 2010) is critical to the future ecology and sustainability of grasslands. Of particular interest, we provide scientific foundation to land managers on ecotype suitability to climate, which is relevant to the USDA Conservation Reserve program (http://www.ks.nrcs.usda.gov/programs/crp/) that restores grasslands on marginal agricultural lands (SCS, 1990). *Andropogon gerardii* is widely used in these conservation plantings throughout the North American central grassland, covering nearly 3 million ha (http://www.fsa.usda.gov). Furthermore, about 60% of total agricultural production in KS (~$10 billion, NASS, 2018) was attributed to cattle production, and *A. gerardii* is the main forage grass for cattle in this region. Our experiment demonstrates that genetic constraint may limit a population's ability to adjust to changing climates. If populations cannot adjust to environmental change through phenotypic plasticity, populations will have to migrate to match their future climate conditions (Smith et al., 2017), or migration will need to be facilitated through human intervention, restoration (Christmas et al., 2016; Nicotra et al., 2010). Thus, knowledge of climate‐matched ecotypes is urgently needed to prevent local extinction in changing climates.

## CONFLICT OF INTEREST

None declared.

## Supporting information

Fig S1‐S6

Table S1‐S11

Supplementary Material

## Data Availability

Data for this study are available at Dryad Digital Repository: https://doi.org/10.5061/dryad.tqjq2bvw5.
