# Supplementary material for: Adaptive genetic potential and plasticity of trait variation in the foundation prairie grass Andropogon gerardii across the US Great Plains’ climate gradient: Implications for climate change and restoration
Source: Evol Appl. 2020 Jun 22;13(9):2333–56. doi: 10.1111/eva.13028 (PMC7513703; doi:10.1111/eva.13028)
Supplement: Supplementary file 1 — Fig S1‐S6 [file EVA-13-2333-s001.pdf]

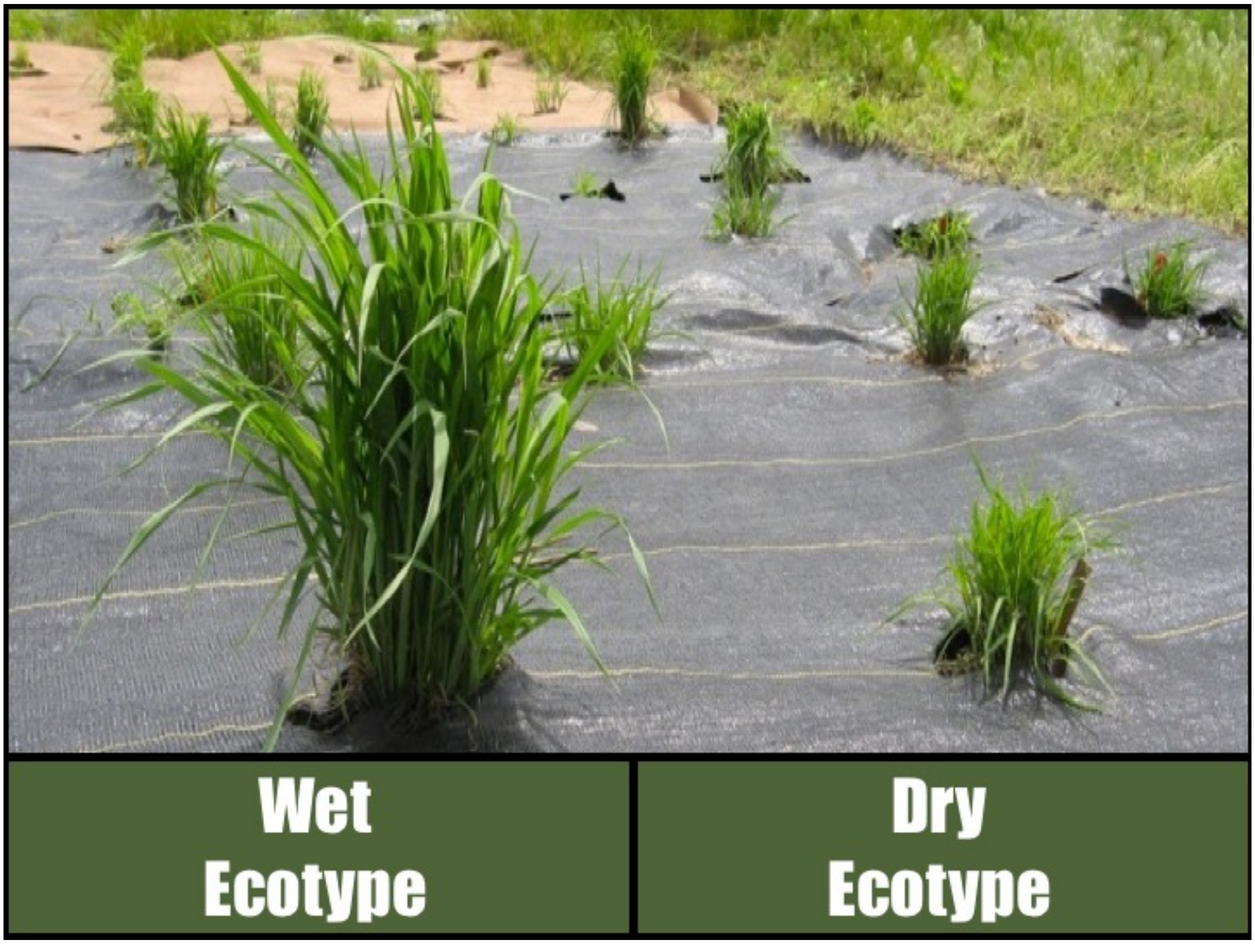

S Fig. 1. Illustrative photograph of morphological differences between a plant from the wet ecotype (forefront, left) and another from the dry ecotype (forefront right). Photo taken in Carbondale Illinois planting site.

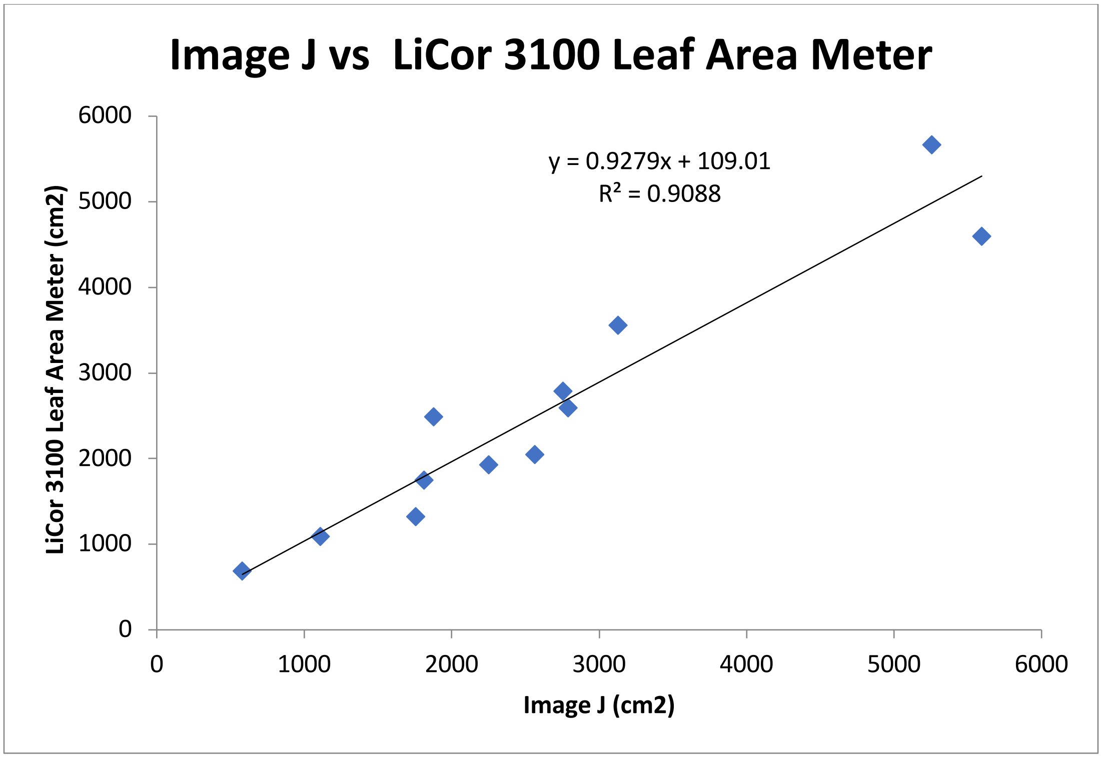

S Fig. 2. Scatterplot and fitted regression line of canopy area measurements obtained from images using ImageJ and from LiCor leaf area meter on the same plant. N=12 plants measured.

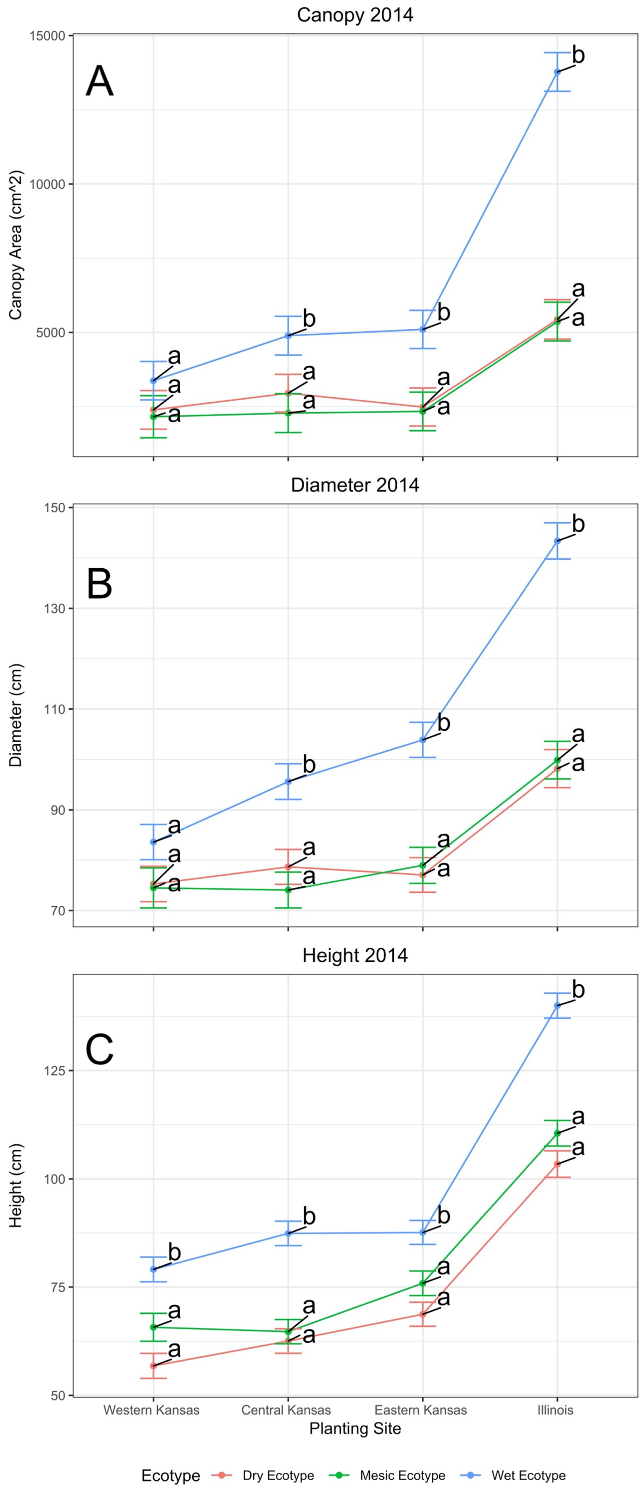

S Fig. 3. Sampling year 2014. Least square mean estimates (± standard error) of Vegetative morphological traits for ecotypes (dry, mesic, wet) across reciprocal garden planting sites for Western KS (Colby KS), Central KS (Hays KS), Eastern KS (Manhattan KS), and Illinois (Carbondale Illinois). A canopy area (cm^2), b) diameter (cm), c) height (cm), d) blade width (mm). Sites with different letters indicate significant differences.

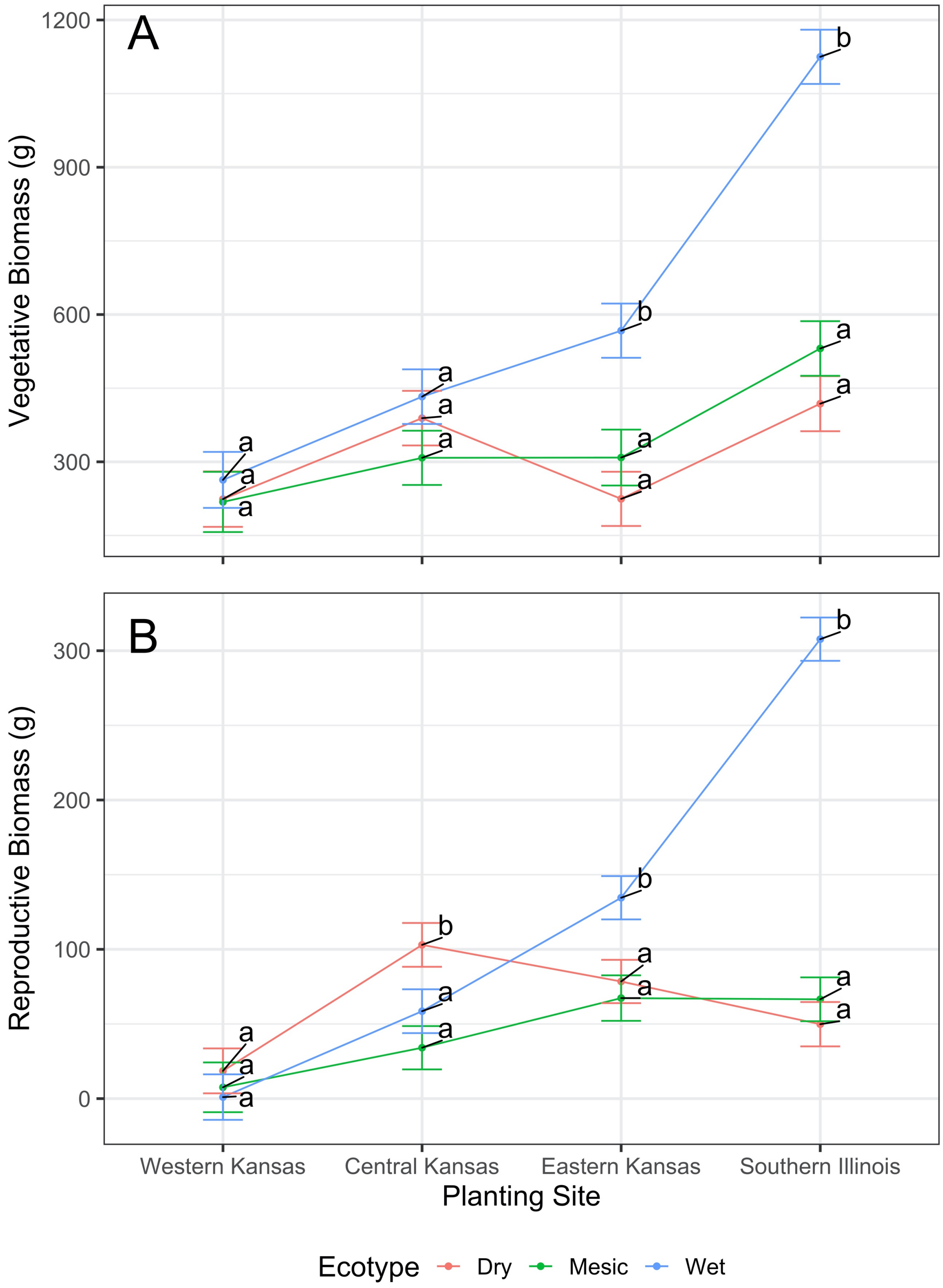

S. Fig. 4. Least square mean estimates (± standard error) for vegetative and reproductive biomass across reciprocal garden planting sites in Western Ks (Colby KS), Central KS (Hays KS), Eastern KS (Manhattan KS), and Illinois (Carbondale Illinois). A) vegetative and B) reproductive. Different letters indicate significant differences among home site ecotypes.

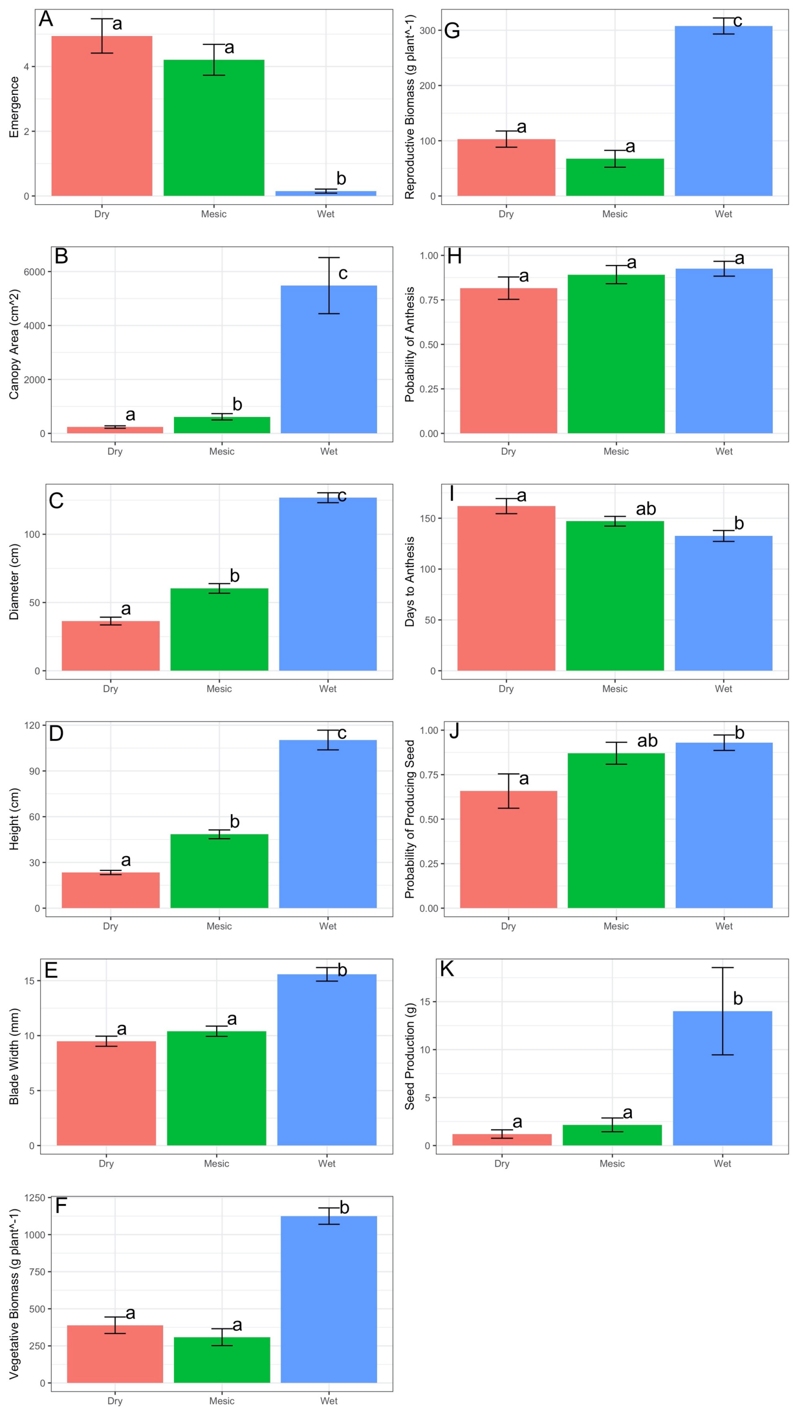

S. Fig. 5. Least square mean estimates for vegetative morphological traits and reproductive fitness traits for ecotypes within their home site. Note that Colby was not included because there was no Eastern KS ecotype. A) canopy area, B) height, C) blade width, D) diameter, E) vegetative biomass, F) reproductive biomass, G) probability of anthesis, H) days to anthesis, I) probability of seed production, J) seed mass. Within a trait, Different letters indicate significant differences among home site ecotypes.

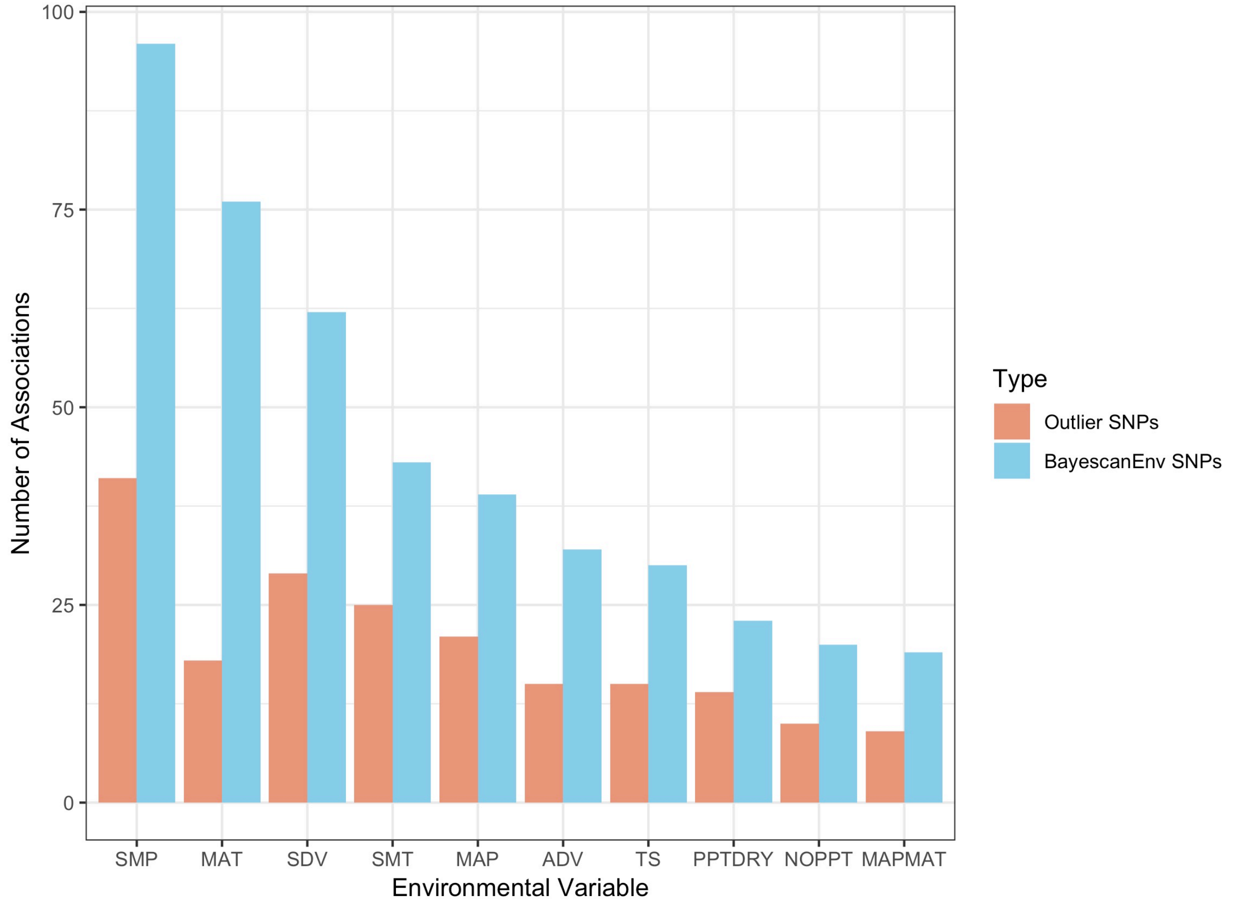

S. Fig. 6. Histogram depicting the number of SNP markers associated with environmental conditions based on BayescanEnv analyses. 440 SNPs (out of a total of 4462) were identified in BayescanEnv as being significantly associated with climate. Approximately 10% (of the 4462) were significantly associated with environmental factors (as defined in table 1) and the number of SNPs occurrences for each environmental variable is denoted by blue bar. (Not that a single SNP can be associated with more than one variable) SNPs were most associated with precipitation variables (20%) and secondarily, with aspects of temperature (mean annual temperature 76, seasonal diurnal variation 60). If one takes all the outliers SNPs identified in Bayescan and Bayenv, 197 SNPs were significantly associated with climate variables (defined in table 1) and the number of SNP occurrences for each environmental variable is denoted by red bar. Outlier SNPs (red bar) were most associated with precipitation variables (20%, or 41), then followed by seasonal mean precipitation followed to a lesser extent by variables related to temperature (seasonal diurnal variation 29, and seasonal annual temperature 25). Climate variable acronyms as in Table 1.
