# Supplementary material for: Adaptive genetic potential and plasticity of trait variation in the foundation prairie grass Andropogon gerardii across the US Great Plains’ climate gradient: Implications for climate change and restoration
Source: Evol Appl. 2020 Jun 22;13(9):2333–56. doi: 10.1111/eva.13028 (PMC7513703; doi:10.1111/eva.13028)
Supplement: Supplementary file 2 — Table S1‐S11 [file EVA-13-2333-s002.pdf]

	Ecotype	Western Kansas	Central Kansas	Eastern Kansas	Southern Illinois
**Emergence (units) 2010**	**Dry**	12.1817 ± 1.1169	***4.9455 ± 0.5306***	4.1009 ± 0.4573	0.6913 ± 0.1437
	**Mesic**	11.9116 ± 1.1679	6.1284 ± 0.6271	***4.2081 ± 0.4766***	0.6732 ± 0.14
	**Wet**	12.2145 ± 1.0912	5.0276 ± 0.5322	4.1392 ± 0.4561	***0.1498 ± 0.06224***
**Canopy (cm^2) 2010**	**Dry**	81.57 ± 15.7679	***235.54 ± 44.9974***	411.4 ± 78.1480	2031.76 ± 385.95
	**Mesic**	195.93 ± 41.3724	314.95 ± 59.6531	***614.58 ± 118.148***	2785.08 ± 529.05
	**Wet**	140.2 ± 26.9490	314.03 ± 59.6531	1181.66 ± 224.47	***5479.18 ± 1040.8***
**Canopy (cm^2) 2014**	**Dry**	2393.83 ± 649.52	***2953.63 ± 634.6***	2490.33 ± 640.44	5437.11 ± 664.7
	**Mesic**	2162.06 ± 709.71	2283.75 ± 652.19	***2341.46 ± 647.35***	5363.91 ± 652.12
	**Wet**	3376.03 ± 647.33	4889.11 ± 652.11	5099.52 ± 642.88	***13775 ± 652.11***
**Diameter (cm) 2010**	**Dry**	24.3974 ± 2.7323	***36.4123 ± 2.8323***	48.1352 ± 3.4505	87.5931 ± 3.6794
	**Mesic**	38.1319 ± 2.9733	44.4393 ± 2.8325	***60.3327 ± 3.5115***	102.30 ± 3.6786
	**Wet**	35.1818 ± 2.6848	50.4293 ± 2.7820	80.2354 ± 3.3977	***126.76 ± 3.6285***
**Diameter (cm) 2014**	**Dry**	75.2644 ± 3.5022	***78.6601 ± 3.4727***	77.0613 ± 3.444	98.1805 ± 3.7798
	**Mesic**	74.4843 ± 3.9772	74.0586 ± 3.5592	***78.9596 ± 3.5917***	99.856 ± 3.7289
	**Wet**	83.5922 ± 3.5037	95.6035 ± 3.5397	103.88 ± 3.4747	***143.37 ± 3.6014***
**Blade Width (mm) 2010**	**Dry**	8.2821 ± 0.4661	***9.4908 ± 0.4607***	11.25 ± 0.458	13.75 ± 0.6169
	**Mesic**	7.3248 ± 0.5099	8.306 ± 0.4607	***10.4016 ± 0.4636***	12.8 ± 0.6169
	**Wet**	8.3354 ± 0.4635	9.95 ± 0.458	11.9 ± 0.458	***15.575 ± 0.6169***
**Height (cm) 2010**	**Dry**	14.301 ± 0.8569	***23.387 ±1.3809***	32.577 ± 1.9097	78.399 ± 4.5961
	**Mesic**	24.078 ± 1.5976	32.284 ± 1.9064	***48.434 ± 2.8822***	82.487 ± 4.8359
	**Wet**	24.312 ± 1.4467	37.074 ± 2.1735	65.175 ± 3.8209	***110.283 ± 6.4654***
**Height (cm) 2014**	**Dry**	56.8105 ± 2.8852	***62.5429 ± 2.8264***	68.725 ± 2.7721	103.43 ± 3.0608
	**Mesic**	65.7187 ± 3.2185	64.7111 ± 2.7989	***75.8707 ± 2.8272***	110.53 ± 2.9517
	**Wet**	79.066 ± 2.8571	87.3967 ± 2.8279	87.6125 ± 2.7721	***140.03 ± 2.8866***
**Vegetative Biomass (g per plant)**	**Dry**	223.98 ± 56.48	***388.94 ± 55.63***	224.53 ± 55.24	418.37 ± 56.04
	**Mesic**	218.27 ± 61.33	308.12 ± 55.24	***308.68 ± 56.94***	530.84 ± 55.63
	**Wet**	263.16 ± 57.03	432.72 ± 55.65	567.14 ± 55.24	***1124.94 ± 55.23***
**Reproductive Biomass (g per plant)**	**Dry**	18.5807 ± 15.0391	***102.97 ± 14.6761***	78.4569 ± 14.505	49.8466 ± 14.853
	**Mesic**	7.5699 ± 16.6620	34.0776 ± 14.505	***67.3245 ± 15.238***	66.5200 ± 14.676
	**Wet**	1.0163 ± 15.2386	58.5502 ± 14.678	134.54 ± 14.5051	***307.73 ± 14.5051***
**Probability of Anthesis 2012**	**Dry**	0.3784 ± 0.07973	***0.8158 ± 0.06289***	0.9487 ± 0.03532	0.9737 ± 0.02597
	**Mesic**	0.03333 ± 0.03277	0.2632 ± 0.07143	***0.8919 ± 0.05105***	0.7838 ± 0.06768
	**Wet**	0.05263 ± 0.03622	0.7895 ± 0.06613	0.9250 ± 0.04165	***0.9250 ± 0.04165***
**Days to Anthesis Julian days 2012**	**Dry**	155.62 ± 4.8113	***161.87 ± 7.4362***	122.35 ± 4.6211	103.89 ± 5.3609
	**Mesic**	-	180.00 ± 12.8923	***147.01 ± 4.7781***	131.16 ± 5.7908
	**Wet**	-	195.22 ± 13.0580	143.88 ± 4.6222	***132.54 ± 5.3620***
**Probability of Seed Production 2012**	**Dry**	0.3580 ± 0.07934	***0.6579 ± 0.09646***	0.9264 ± 0.04467	0.8713 ± 0.06134
	**Mesic**	8.117E-9 ± 0.000017	0.1485 ± 0.06487	***0.8703 ± 0.06161***	0.7380 ± 0.08700
	**Wet**	7.725E-9 ± 0.000014	0.1463 ± 0.06566	0.9068 ± 0.05035	***0.9296 ± 0.04340***
**Seed Production (g per plant) 2012**	**Dry**	1.1704 ± 0.5601	***1.2018 ± 0.4397***	4.2709 ± 1.3739	4.3822 ± 1.4588
	Mesic	-	0.3467 ± 0.2648	***2.1576 ± 0.7191***	2.7106 ± 0.9565
	Wet	-	0.2481 ± 0.1982	7.3587 ± 2.3897	***14.0057 ± 4.5491***

S Table 1. Estimated least square means and corresponding SE of vegetative, biomass, and reproductive response variables by ecotype for each planting site. Note: Home site in bold italics

	Site		Ecotype		Site x Ecotype		Time		Site x Time		Ecotype x Time		Site x Ecotype x Time	
Variable	$F_{Df\,n, Df\,d}$	P	$F_{Df\,n, Df\,d}$	P	$F_{Df\,n, Df\,d}$	P	$F_{Df\,n, Df\,d}$	P	$F_{Df\,n, Df\,d}$	P	$F_{Df\,n, Df\,d}$	P	$F_{Df\,n, Df\,d}$	P
Canopy Area (2010, 2014)	$213.33_{3, 36.26}$	**<0.0001**	$14.63_{2, 9.1}$	**0.0015**	$5.33_{6, 805.6}$	**<0.0001**	$1204.87_{1, 795.2}$	**<0.0001**	$90.44_{3, 795.2}$	**<0.0001**	$11.48_{2, 795.2}$	**<0.0001**	$1.50_{6, 795.2}$	**0.01738**
Diameter (2010, 2014)	$270.35_{3, 33.27}$	**<0.0001**	$3.66_{2, 8.95}$	0.0688	$4.96_{6, 803.5}$	**<0.0001**	$426.70_{1, 793.4}$	**<0.0001**	$36.65_{3, 793.4}$	**<0.0001**	$18.04_{2, 793.4}$	**<0.0001**	$4.38_{6, 793.4}$	**0.0002**
Height (2010, 2014)	$108.68_{3, 37.53}$	**<0.0001**	$24.00_{2, 8.1}$	**0.0004**	$27.85_{6, 801.1}$	**<0.0001**	$108.68_{1, 792.8}$	**<0.0001**	$12.31_{3, 792.8}$	**<0.0001**	$39.99_{2, 792.8}$	**<0.0001**	$5.04_{6, 792.8}$	**<0.0001**

	Site		Ecotype		Site x Ecotype	
Emergence	$218.84_{3, 28.73}$	**<0.0001**	$3.80_{2, 22.3}$	**0.0380**	$2.81_{6, 439}$	**0.0107**
Blade Width (2010)	$94.16_{3, 37.1}$	**<0.0001**	$5.33_{2, 10}$	**0.0264**	$1.01_{6, 331}$	0.4162
Vegetative Biomass (2011)	$13.31_{2, 9}$	**0.0020**	$62.86_{3, 36}$	**<0.0001**	$16.01_{6, 385}$	**<0.0001**
Reproductive Biomass (2011)	$31.15_{2, 9}$	**<0.001**	$36.70_{3, 36}$	**<0.0001**	$27.61_{6, 385}$	**<0.0001**
Probability of Anthesis (2012)	$29.82_{3, 27}$	**<0.0001**	$10.56_{2, 9}$	**0.0044**	$1.68_{6, 27}$	0.1649
Days to Anthesis (2012)	$41.22_{3, 27}$	**<0.0001**	$9.13_{2, 17.6}$	**0.0019**	$0.47_{4, 27}$	0.7543
Probability of Seed Production (2012)	$42.17_{2, 27}$	**<0.0001**	$3.10_{2, 7.7}$	0.1028	$3.07_{4, 27}$	**0.0330**
Seed Production (2012)	$17.31_{3, 44.57}$	**<0.0001**	$3.52_{2, 13.6}$	0.0587	$3.53_{4, 223.9}$	**0.0081**

S Table 2. Statistical analyses of response variables from 2010, 2011, 2012 and 2014. Factors include planting site (S), Ecotype (E), and S X E interactions. Upper table; Variables with multiple years of measurements (Canopy Area, Diameter, and Height). Lower table Variables with only one year of measurements (Blade width, Vegetative biomass, Reproductive biomass, Probability of anthesis, Days to anthesis, Probability of seed production, and Seed production). For variables measures across two years (2010 and 2014 for canopy area, diameter, and height) the full three-way interaction with Time (T) is provided.

	Dry Ecotype	Mesic Ecotype	Wet Ecotype
Dry Ecotype	33 (31.1%)	1 (0.9%)	0
Mesic Ecotype	5 (4.7%)	30 (28.3%)	0
Wet Ecotype	0	0	37 (34.9%)

S Table 3. Table showing the classification rates of the 10-fold cross validation. Note that 94.3% are correctly assigned (data from along the diagonal). Total of 106 individuals analyzed with 34 dry, 35 mesic, and 37 wet ecotypes.

Parameter Estimates Principle Coordinate 1					
Parameter	DF	Estimate	Standard Error	t-value	p-value
Intercept	1	-18.83779	2.902155	-6.49	1.0000
Annual Diurnal Variation	1	1.614488	0.196429	8.22	0.0118
Number of Precipitation Events > 1.25 cm	1	-0.255112	0.025752	-9.91	0.0010
Seasonal Mean Precipitation	1	0.081677	0.021503	3.8	<.0001
Seasonal Mean Temperature	1	0.404559	0.086862	4.66	<.0001
Seasonal Diurnal Variation	1	-0.6459	0.213481	-3.03	0.0032

S Table 4. Final model obtained from stepwise regression depicting selected environmental conditions considered to be significantly associated with morphology PC score 1 for ecotypes grown in their home site

Parameter Estimates Principal Coordinate 1					
Parameter	DF	Estimate	Standard Error	t-value	p-value
Intercept	1	8.569889	1.465610	5.85	1.0000
Mean Annual Precipitation	1	-0.381848	0.022347	-17.09	<.0001
Seasonal Mean Precipitation	1	0.496011	0.055912	8.87	<.0001
Parameter Estimates Principal Coordinate 2					
Parameter	DF	Estimate	Standard Error	t-value	p-value
Intercept	1	-15.448569	3.852113	-4.01	1.0000
Annual Mean Temperature	1	-1.540529	0.435119	-3.54	0.0005
Mean Annual Precipitation	1	-0.231000	0.024784	-9.32	<.0001
Number of Precipitation Events > 1.25 cm	1	1.035900	0.095066	10.90	<.0001
Seasonal Mean Temperature	1	1.509246	0.240293	6.28	<.0001

S Table 5. Final model obtained from stepwise regression depicting selected environmental conditions considered to be significantly associated with genetic distance principal coordinates 1 and 2 for ecotypes grown in their home site. Adjusted R^2 PCo1 = 0.5593 and Adjusted R^2 PCo2 = 0.3936.

12MI (Wet)	DES (Wet)	FUL (Wet)	WAL (Wet)	KON (Mesic)	TAL (Mesic)	TOW (Mesic)	CAR (Mesic)	CDB (Dry)	REL (Dry)	SAL (Dry)	WEB (Dry)	
0.000												12MI
0.023	0.000											DES
0.020	0.022	0.000										FUL
0.018	0.021	0.020	0.000									WAL
0.029	0.034	0.026	0.028	0.000								KON
0.026	0.029	0.023	0.025	0.014	0.000							TAL
0.032	0.035	0.028	0.030	0.011	0.014	0.000						TOW
0.024	0.028	0.021	0.022	0.013	0.014	0.013	0.000					CAR
0.033	0.035	0.029	0.032	0.012	0.017	0.012	0.014	0.000				CDB
0.030	0.033	0.027	0.028	0.012	0.016	0.011	0.013	0.012	0.000			REL
0.024	0.028	0.021	0.023	0.012	0.014	0.012	0.011	0.013	0.011	0.000		SAL
0.033	0.037	0.029	0.033	0.012	0.016	0.011	0.014	0.011	0.012	0.013	0.000	WEB

S Table 6. Pairwise F_{st} among pairs of populations.

Footnote: Pairwise F_{st} between ecotypes: Wet-Mesic = 0.0275, Standard Deviation = 0.004; Wet-Dry = 0.0297, Standard Deviation = 0.004; and Mesic-Dry = 0.013, Standard Deviation = 0.002.

Analysis	Markers	Chr	Position	Gene Id	Gene Information
Bayescan	TP3814	2	56601981	Sb02g023140.1	PF07732,PF00394,PF07731 PTHR11709 KOG1263 AT4G39830.1 L-ascorbate oxidase, putative
Bayescan	TP23759	2	53236055	Sb02g021490.1	PF03055 PTHR10543 KOG1285 AT3G63520.1 CCD1 CCD1 (CAROTENOID CLEAVAGE DIOXYGENASE 1); 9-cis-epoxycarotenoid dioxygenase
Bayescan	TP27974	6	39909469	Sb06g014410.1	PF02416 AT5G52440.1 HCF106 HCF106; proton motive force dependent protein transmembrane transporter
Bayescan	TP63759	1	55492323	Sb01g032580.1	PF03195 AT1G67100.1 LBD40 LBD40 (LOB DOMAIN-CONTAINING PROTEIN 40)
Bayescan	TP69704	10	42173347	Sb10g019720.1	PF02453 PTHR10994 KOG1792 AT4G11220.1 BTI2 BTI2 (VIRB2-INTERACTING PROTEIN 2)
Bayescan	TP70880	9	47608900	Sb09g019060.1	PF02182,PF05033,PF00856 PTHR22884 KOG1082 AT5G13960.1 SUVH4 SUVH4 (SU(VAR)3-9 HOMOLOG 4); double-stranded methylated DNA binding / histone methyltransferase(H3-K9 specific) / methyl-CpG binding / methyl-CpNpG binding / methyl-CpNpN binding
Bayescan	TP79982	3	71975298	Sb03g044630.1	PF02362,PF06507 AT5G62000.1 ARF2 ARF2 (AUXIN RESPONSE FACTOR 2); protein binding / transcription factor
Bayescan	TP90370	3	6472172	Sb03g006365.1	PTHR23273 KOG0851 AT4G19130.1 DNA binding / nucleic acid binding / zinc ion binding
Bayescan	TP100780	5	5098115	Sb05g004185.1	PF03140 AT3G44710.1 unknown protein
Bayescan	TP105473	2	77853480	Sb02g044040.1	PF02178,PF00855 AT3G05430.1 PWWP domain-containing protein
Bayescan	TP110077	8	1046928	Sb08g001115.1	PF02493,PF01504 PTHR23086 KOG0229 2.7.1.68 K00889 AT3G07960.1 phosphatidylinositol-4-phosphate 5-kinase family protein
Bayescan	TP119018	1	60290323	Sb01g036670.1	PF00046 PTHR19418 AT3G03660.1 WOX11 WOX11 (WUSCHEL related homeobox 11); DNA binding / transcription factor
Bayescan	TP150845	4	61081684	Sb04g031080.1	PF03169 PTHR22601 KOG2262 AT5G64410.1 OPT4 OPT4 (OLIGOPEPTIDE TRANSPORTER 4); oligopeptide transporter
Bayescan	TP152852	2	77115416	Sb02g043300.1	PF00046 PTHR19418:SF56 AT2G33880.1 HB-3 HB-3; transcription factor
Consensus	TP97659	1	26916072	Sb01g021990.1	PF01397,PF03936 5.5.1.13 K04120 AT4G02780.1 GA1 GA1 (GA REQUIRING 1); ent-copalyl diphosphate synthase/ magnesium ion binding
Consensus	TP22546	3	10907551	Sb03g010110.1	PF00097 AT1G75400.1 protein binding / zinc ion binding
Consensus	TP48670	7	18950899	Sb07g010450.1	PF00141 1.11.1.7 K00430 AT4G16270.1 peroxidase 40 (PER40) (P40)
Consensus	TP14132	3	59665754	Sb03g031310.1	PF00310,PF04898,PF01645,PF01493,PF07992,PF00070 PTHR11938 KOG0399 1.4.1.13 K00265 AT5G53460.1 GLT1 GLT1; glutamate synthase (NADH)
Bayenv2	TP171997	7	11673324	Sb07g007530.1	PF01263 PTHR11122 KOG1594 AT5G57330.1 aldose 1-epimerase family protein
Bayenv2	TP4696	1	73079404	Sb01g050120.1	PTHR10343 AT3G52180.1 SEX4 SEX4 (STARCH-EXCESS 4); polysaccharide binding / protein tyrosine/serine/threonine phosphatase
Bayenv2	TP103404	3	52366325	Sb03g026040.1	PF00160 PTHR11071 KOG0885 5.2.1.8 K01802 AT4G33060.1 peptidyl-prolyl cis-trans isomerase cyclophilin-type family protein
Bayenv2	TP16994	9	51311839	Sb09g021780.1	PF02365 AT5G22380.1 anac090 anac090 (Arabidopsis NAC domain containing protein 90); transcription factor
Bayenv2	TP23740	2	67684318	Sb02g033050.1	AT5G42330.1 unknown protein
Bayenv2	TP63473	4	57503730	Sb04g027590.1	PF00191 PTHR10502 KOG0819 AT5G10230.1 ANNAT7 ANNAT7 (ANNEXIN ARABIDOPSIS 7); calcium ion binding / calcium-dependent phospholipid binding
Bayenv2	TP107489	1	47278328	Sb01g027420.1	PF05770 AT4G33770.1 inositol 1,3,4-trisphosphate 5/6-kinase family protein
Bayenv2	TP68168	9	188110	Sb09g000390.1	PF00023,PF07719,PF00515 PTHR18958 KOG0548 AT3G04710.1 ankyrin repeat family protein

S Table 7. Candidate gene annotations from Bayescan analysis (50), the top 1% of Bayenv2 outlier analysis (46), and the consensus markers (15) between the two analyses. Identification is from mapping big bluestem reads to the sorghum genome to find the position. Table shows identifies how the gene was found, using Bayenv or Bayescan or both (Analysis), big bluestem marker id (Markers), to which sorghum chromosome the marker maps (Chr) position on that chromosome (Position), gene identifier of the gene near the sorghum position (Gene Id), and the annotation information from that gene (Gene Information).

Footnote: Bayenv outliers lacking annotation included TP67155, TP505, TP125027, TP39152, TP168589, TP35977, TP61552, TP29318, TP119835, TP123355, TP69553, TP143151, TP171774, TP105122, TP147959, TP132908, TP103399, TP156046, TP168955, Both Bayenv and Bayescan TP1712, TP114252, TP118569, TP5219, TP62618, TP19463, TP52052, TP27663, TP104813, TP34353, TP47256; Bayescan TP24620, TP25391, TP27700, TP28582, TP38460, TP48595, TP49355, TP61449, TP77269, TP79145, TP91916, TP95678, TP97404, TP97704, TP129182 , TP124284, TP144242, TP161527, TP164302, TP172967, TP173308

Marker Number	SMP	MAT	SDV	SMT	MAP	ADV	TS	PPTDRI	NOPPT	MAP/MAT
142	-	-	0.0010002	-	-	-	-	-	-	-
392	-	-	0.019604	-	0.017604	0.013203	-	-	-	-
429	-	-	-	-	-	-	-	-	-	-
478	0.005201	-	0.015403	0.014003	-	-	-	-	-	-
561	-	-	0.0056011	-	-	-	-	-	-	-
730	0.00040008	-	0.026605	-	-	-	-	-	-	-
752	0.0026005	-	-	0.010802	-	-	-	-	-	-
789	0.026205	-	0.005001	0.014403	-	-	-	-	-	-
919	0.014803	-	-	-	-	-	-	-	-	-
929	0.0018004	-	-	0.0054011	0.013003		-	-	-	-
969	0.00080016	-	0.0038008	0.016603	0.0030006	0.0044009	0.0044009	0.011602	0.0084017	0.0086017
999	0.00080016	0.00080016	0.0020004	***0.027806***	0.0066013	0.0086017	0.013603	0.016203	0.015803	***0.021204***
1124	0.00020004	-	-	-	-	-	-	-	-	-
1186	-	-	0.0026005	-	0.0018004	0.0040008	0.0012002	0.0016003	**0.023405**	0.011202
1219	0.0028006	-	-	-	-	**0.021404**	-	-	-	-
1227	-	0.00080016	0.021604	0.00040008	0.0020004	-	0.014203	-	***0.023805***	0.013603
1252	0.00020004	-	-	0.0028006	0.00080016	0.00020004	0.0012002	0.00080016	0.0018004	0.00060012
1272	0.0094019	-	-	-	-	-	-	-	-	-
1341	0.00040008	0.00080016	0.0012002	0.0030006	0.020604	-	-	**0.020804**	-	-
1573	-	-	-	-	-	-	-	-	-	-
1620	0.0024005	-	0.0022004	-	0.011602	0.0074015	0.0034007	0.010802	-	-
1764	0.012803	-	-	-	-	-	-	-	-	-
1781	-	0.00040008	0.011202	0.011202	-	-	-	-	-	-
1857	***0.030206***	-	-	-	-	-	-	-	-	-
2065	0.023605	-	-	-	-	-	-	-	-	-
2072	0.016003	-	-	-	-	-	-	-	-	-
2205	0.0072014	-	-	0.0026005	***0.024805***	-	-	-	-	-
2354	-	-	-	-	-	-	-	-	-	-
2438	-	-	-	-	-	-	-	-	-	-
2522	-	-	0.011802	-	-	-	-	-	-	-
2598	-	-	-	-	-	-	-	-	-	-
2754	0.010402	-	0.0022004	-	-	-	-	-	-	-
2788	0.014203	-	0.0060012	0.0060012	-	-	-	-	-	-
2792	-	-	0.012402	-	-	-	-	-	-	-
2912	-	0.00060012	0.00020004	0.013803	-	-	0.0030006	0.0036007	0.0012002	0.0024005
2921	-	-	-	-	-	0.00060012	-	-	-	-
3060	0.010802	0.0046009	-	-	-	-	-	-	-	-
3146	-	-	-	-	-	-	-	-	-	-
3167	0.0016003	0.0030006	0.021204	0.0074015	0.022204		-	-	-	-
3302	-	-	-	0.012402	0.00040008	0.0022004	0.0044009	0.0054011	-	0.012002
3305	-	-	-	-	-	-	-	-	-	-
3402	0.0036007	-	-	0.005201	0.015203	-	-	-	-	-
3435	0.0036007	0.00060012	-	-	-	-	-	-	-	-
3438	0.0074015	-	-	0.0022004	-	-	-	-	0.013403	-
3482	0.0012002	0.00020004	***0.028606***	**0.025405**	0.0076015	-	-	-	-	-
3514	-	0.0030006	0.0020004	-	0.00040008	0.0028006	0.00020004	0.0024005	0.0038008	0.0012002
3536	0.024405	-	-	-	-	-	-	-	-	-
3567	-	0.00020004	-	-	0.020804	-	-	-	-	-
3587	-	0.0024005	0.00060012	0.0028006	0.013403	0.004801	0.013603	0.018204	-	-
3781	0.0022004	0.0020004	0.022605	-	0.0034007	-	0.010002	-	0.0058012	**0.013803**
3806	0.00080016	-	-	-	-	-	-	-	-	-
3935	0.0020004	0.0026005	-	-	-	-	-	-	-	-
4008	0.0054011	-	0.025405	-	**0.023805**	-	-	-	-	-
4057	0.00080016	-	0.0034007	0.010802	-	-	-	0.016203	-	-
4189	0.0038008	-	-	0.00060012	-	-	-	-	-	-
4268	-	0.0022004	0.0090018	0.0014003	0.013803	0.004801	**0.016203**	***0.021604***	-	-
4349	0.0062012	***0.014803***	-	0.0014003	-	-	-	-	-	-
4371	0.0018004	**0.0056011**	0.024205	0.00020004	-	***0.022204***	-	-	-	-
4451	0.00040008	0.00040008	-	0.0032006	-	-	-	-	-	-
4464	-	-	-	-	-	-	-	-	-	-
4470	0.0020004	0.0024005	**0.028006**	-	0.0062012	-	0.012402	-	0.0068014	-
4513	0.0040008	-	0.00040008	-	-	0.011402	0.014003	0.0046009	-	-
4560	0.019404	-	-	-	-	0.016603	***0.019604***	0.011202	-	-
4573	**0.030206**	-	-	-	-	-	-	-	-	-

S Table 8. *Bayscenv* climate association with SNPs and 11 climate variables. For each environmental variable (column), the most strongly associated SNP is indicated with bold italics and underlined, and the second strongest association is indicated with only bold. Only significant associations between outliers and environmental variables are presented here (q-value < 0.05), values indicate estimates of the strength of selection for a given loci with each climate variable (dashes indicate nonsignificant associations).

**Variation explained**	**Model 1 (Full)** Joint Effect of Geography and Climate	**Model 2** Climate Conditioned by Geography	**Model 3** Geography conditioned by Climate
Constrained (total explained)	863	642 (74%)	129 (15%)
Total joint explained	771 (89%)		
Total unexplained	92 (11%)		
Total variation	18710	18710	18710

STable 9. RDA results for 9 climate variables (Elevation, Mean Annual Precipitation, Seasonal Mean Precipitation, Annual Diurnal Variation, Seasonal Diurnal Variation, Mean Annual Temperature, Temperature Severity Index, and Mean Annual Precipitation/Mean Annual Temperature) and geographic distance (Latitude and Longitude). Table shows the partitioning of variation between the full RDA (model 1) including the joint effects of both geography and climate, the partial RDA model 2 including the effects of climate conditioned by geography and the partial RDA model 3 including the effects of geography conditioned by climate.

	**RDA1**	**RDA2**
Latitude	0.7916	0.17256
Longitude	-0.7863	-0.57245
Mean Annual Precipitation	-0.7788	-0.52479
Mean Annual Precipitation/ Mean Annual Temperature	-0.7609	-0.54181
Elevation	0.7464	0.54051
Temperature Severity Index	0.7	0.60288
Mean Annual Temperature	-0.5581	-0.42841
Annual Diurnal Variation	0.593	0.70931
Seasonal Diurnal Variation	0.4848	0.61572
Mean Seasonal Precipitation	-0.433	-0.49167
Mean Seasonal Precipitation	-0.2179	-0.07754

S Table 10. Geographic and climate loading scores for RDA1 and RDA2 for the pRDA model including the joint effect of geography and climate. Precipitation of the driest year and number of precipitation events was excluded due to collinearity.

Variable	Markers	Chromosome #	Position	Gene Id	Gene Information
**Height**	TP14132	Chromosome_3	59665754	Sb03g031310.1	PF00310,PF04898,PF01645,PF01493,PF07992,PF00070 PTHR11938 KOG0399 1.4.1.13 K00265 AT5G53460.1 GLT1 GLT1; glutamate synthase (NADH)
**Height**	TP152852	Chromosome_2	77115416	Sb02g043300.1	PF00046 PTHR19418:SF56 AT2G33880.1 HB-3 HB-3; transcription factor
**Height**	TP22546	Chromosome_3	10907551	Sb03g031310.1	PF00097 AT1G75400.1 protein binding / zinc ion binding
**Height**	TP48670	Chromosome_7	18950899	Sb07g010450.1	PF00141 1.11.1.7 K00430 AT4G16270.1 peroxidase 40 (PER40) (P40)
**Height**	TP70880	Chromosome_9	47608900	Sb09g019060.1	PF02182,PF05033,PF00856 PTHR22884 KOG1082 AT5G13960.1 SUVH4 SUVH4 (SU(VAR)3-9 HOMOLOG 4); double-stranded methylated DNA binding / histone methyltransferase(H3-K9 specific) / methyl-CpG binding / methyl-CpNpG binding / methyl-CpNpN binding
**Height**	TP97659	Chromosome_1	26916072	Sb01g021990.1	PF01397,PF03936 5.5.1.13 K04120 AT4G02780.1 GA1 GA1 (GA REQUIRING 1); ent-copalyl diphosphate synthase/ magnesium ion binding
**Canopy Area**	TP63759	Chromosome_1	55492323	Sb01g032580.1	PTHR10887 KOG1801 AT1G65810.1 tRNA-splicing endonuclease positive effector-related
**Canopy Area**	TP70880	Chromosome_9	47608900	Sb09g019060.1	PF02182,PF05033,PF00856 PTHR22884 KOG1082 AT5G13960.1 SUVH4 SUVH4 (SU(VAR)3-9 HOMOLOG 4); double-stranded methylated DNA binding / histone methyltransferase(H3-K9 specific) / methyl-CpG binding / methyl-CpNpG binding / methyl-CpNpN binding
**Emergence**	TP119018	Chromosome_1	60290323	Sb01g036670.1	PF00046 PTHR19418 AT3G03660.1 WOX11 WOX11 (WUSCHEL related homeobox 11); DNA binding / transcription factor
**Emergence**	TP70880	Chromosome_9	47608900	Sb09g019060.1	PF02182,PF05033,PF00856 PTHR22884 KOG1082 AT5G13960.1 SUVH4 SUVH4 (SU(VAR)3-9 HOMOLOG 4); double-stranded methylated DNA binding / histone methyltransferase(H3-K9 specific) / methyl-CpG binding / methyl-CpNpG binding / methyl-CpNpN binding
**Blade Width**	TP171997	Chromosome_7	11673324	Sb07g007530.1	PF01263 PTHR11122 KOG1594 AT5G57330.1 aldose 1-epimerase family protein
**Blade Width**	TP26923	Chromosome_3	73373694	Sb03g046210.1	PF00036 PTHR10891 KOG0027 AT1G76640.1 calmodulin-related protein, putative
**Blade Width**	TP124709	Chromosome_6	58175486	Sb06g029600.1	PF01370 PTHR10366 KOG1502 1.3.1.77 K08695 AT1G61720.1 BAN BAN (BANYULS); oxidoreductase
**Diameter**	TP144464	Chromosome_6	44415397	Sb06g016010.1	PF06258 AT5G22350.1 ELM1 ELM1 (ELONGATED MITOCHONDRIA 1)
**Diameter**	TP69676	Chromosome_2	27812706	Sb02g014480.1	PF07719,PF00515,PF00564 PTHR22904 KOG4151 AT2G25290.1 binding

STable 11. SNPs associated with response variables based on *TASSLE* analyses. Total number of SNPs associated = 173 (anthesis 11, height 87, canopy area 28, blade width 7, emergence 38, diameter 2). Of those SNPS that were associated in *TASSLE* and also *Bayescan* outliers, there were a total of 33 (anthesis 1, height 11, canopy area 9, blade width 0, emergence 12, and diameter 0). Only those associations with annotated SNPs are shown here. Gray shading indicates the GA1 SNP.

Markers that were associated, but not annotated: TP118569, TP19463, TP129182, TP14132, TP164302, TP19463, TP25391, TP27700, TP34353, TP47256, TP48595, TP52052, TP61449, TP62618, TP79145, TP91916, TP40577, TP53225, TP64928, TP82066, TP93930, TP106232, TP107688, TP124841, TP125449, TP126048, TP147316, TP153041, TP174440, TP177326, TP15153, TP70053,, TP7498, TP106075, TP140561, TP68489, TP135366, TP169807, TP12076, TP162226, TP44305, TP100504, TP49032, TP101481, TP135939, TP123320, TP18961, TP163294, TP138332, TP143687, TP505, TP180468, TP160634, TP156386, TP151095, TP164220, TP29647, TP117156, TP97430, TP62307, TP86868, TP106592, TP100082, TP144170, TP24765, TP102312, TP113188, TP71544, TP148121, TP34326, TP131010, TP129945, TP80136, TP160911, TP125027, TP93235, TP132908, TP104813, TP114252, TP19463, TP34353, TP52052, TP62618, TP79145, TP53225, TP93930, TP169807, TP82066, TP103399, TP124841, TP135366, TP107688, TP174440, TP138332, TP147316, TP106232, TP177326, TP7498, TP106075, TP162226, TP173892, TP92195, TP104813, TP114252, TP161527, TP19463, TP34353, TP47256, TP52052, TP61449, TP62618, TP79145, TP82066, TP124841, TP177326, TP87444, TP53225, TP70053, TP68489, TP107688, TP93930, TP156386, TP174440, TP147316, TP113188, TP40577, TP44305, TP7498, TP106232, TP18961, TP160372, TP2227, TP30673, TP140561, TP97430, TP124841, TP22543, TP160634, TP174440.
