# Supplementary material for: Adaptive genetic potential and plasticity of trait variation in the foundation prairie grass Andropogon gerardii across the US Great Plains’ climate gradient: Implications for climate change and restoration
Source: Evol Appl. 2020 Jun 22;13(9):2333–56. doi: 10.1111/eva.13028 (PMC7513703; doi:10.1111/eva.13028)
Supplement: Supplementary file 3 — Supplementary Material [file EVA-13-2333-s003.pdf]

Random forest prediction of ecotypes

Random forests were used to test classification of ecotypes based on morphological traits in home site (Breiman 2001). Random forests use an ensemble method (Altman and Krzywinski 2017) for classification based on morphological traits and operates by constructing many decision trees at training and taking a weighted vote of predictions from these trees for final prediction, in our case, ecotype. Phenotypic traits to train and cross validate a random forest predictor model implemented in the *randomForest* R package (Liaw and Wiener 2002).

Seven morphological and reproductive traits (canopy area, height, blade width, diameter, seed production, days to emergence, and days to anthesis) from 106 individual plants growing in their respective home sites (34 from the dry ecotype, 35 from the mesic ecotype, and 37 from the wet ecotype) were used to train and cross validate a random forest model to predict ecotype, implemented in the *randomForest* R package (Liaw and Wiener 2002). For plants that did not flower, the flowering time was included as one week past the date of last observance to prevent missing data. The random forest used morphological traits as predictor variables at each split of decision trees and generated 500 trees for each forest. To assess predictive ability of the random forest model we used a 10-fold cross validation. Individuals within ecotype were randomly assigned to 10 nearly equal nonoverlapping groups or folds. Each one of these 10 folds was used once as a validation set. For folds set for validation, the ecotype designation was masked. Ecotype assignment was predicted using estimates from the seven traits from the remaining data folds (training folds). This was repeated until each of the 10 folds were used as validation once. Individuals were classified to the ecotype bin based on greatest number of votes for that

ecotype across all 500 trees. Assignment of the masked individuals from the validation folds were compared to the true identity of plants to generate misclassification rates and provide a metric of how accurately we can predict ecotypes based on their combination of morphological traits.

II. DNA collection, preparation and analyses

Sample collection. Leaf samples were collected from all individuals with known population origin from the single spaced plants reciprocal gardens across Hays and Manhattan, KS and Carbondale, IL (not Colby). Each site contains 10 replicated blocks with each of the 12 populations represented once in each block. Total number of plants genotyped from the single spaced plants resulted in 110 individuals from the dry ecotype, 106 for the mesic ecotype, and 98 from wet ecotype, all spaced equally amongst the 12 populations for a total of 314 plants. In each site ~100 mg of leaf tissue was collected directly into 96-deep well matrix plates on ice. With the samples being genotyped, all ecotypes were contained within the same plates within each site. Plates collected in the Manhattan planting site were immediately freeze dried. Collections from the Colby and Hays, KS and Carbondale, IL sites were kept frozen on dry ice until freeze-drying. All samples were subsequently ground to a fine powder and stored at -80°C until DNA isolation. We analyzed all plants in the single spaced plots for ploidy level and, they were 6x for wet and dry ecotypes, and mostly 6X for the mesic ecotype with some aneuploids (unpublished data). Thus, results are dependent on other factors, besides ploidy level.

DNA Isolation, library preparation, and sequencing. DNA was isolated using a modified CTAB protocol (Doyle and Doyle 1987) to yield high quality DNA from field grown *A. gerardii* leaves. We used 550 µl per sample of extraction buffer added to ground samples containing 100 mM Tris-HCl, pH 8.0, 100 mM EDTA, pH 8.0, 1.4 M NaCl, 2% CTAB, 12 mM TCEP (a non-toxic substitute for beta-mercaptanethanol), 16 mM Sodium Diethyldithiocarbamate Trihydrate, 4% PVPP. This was followed by 50 µl per sample of reducing buffer containing 100 mM Tris-HCl, pH 8.0, with 120 mM TCEP and 160 mM DIECA added separately. Quality was visually assessed on an 0.8% agrose gel and quantified for DNA concentration with PicoGreen (ThermoFisher.com) and normalized for library preparation. All samples were prepared for genotyping-by-sequencing (Poland and Rife 2012, Elshire *et al.*, 2011) using a two restriction enzyme pair of PstI and MspI. For each plate, sequencing design included barcoding each unique individual and each plate multiplexed (96-plexing) for 200 base paired-end sequencing in one lane per library on an Illumina HiSeq 2000. Ecotypes were split across plates and sequencing runs to avoid sequencing bias.

SNP calling UNEAK pipeline

A SNP calling protocol was applied to genotype single-spaced plants of known ecotype. Individuals were genotyped and SNPs discovered using UNEAK pipeline implemented in TASSEL 3.0 Standalone Pipeline (Lu *et al.* 2013, http://www.maizegenetics.net/tassel). The pipeline is developed for SNP calling in non-model organisms without a reference genome and of any ploidy level. Although *A. gerardii* is being sequenced by JGI, the reference genome is not available yet. Libraries were de-multiplexed from individual

specific barcodes and all reads were confirmed to contain PstI-MspI cut sites. Reads were trimmed to 64 base pairs long and combined into identical tags with five reads required to be retained. Tag pairs containing a single mismatch are combined as putative SNPs with error tolerance of 0.03. Tags were combined across all individuals with minor allele frequency of 0.05. Final SNPs were then filtered two-fold. First, only SNPs were retained that had 6x coverage. Second, for loci that were missing SNPs for >10% of individuals, the entire loci were removed from the final dataset. The final genotype data set resulted in 4,641 high quality SNPs across 314 individuals from single-spaced plants. Following SNP calling, genotyped markers were aligned and anchored, using the BWA-MEM algorithm in the *BWA* software package (Li and Durbin 2009), to the *Sorghum bicolor* genome, one of the closest fully sequenced relatives. This allowed for inference on genomic region of markers within 20kb of candidate genes.

Outlier analysis and association with climate variables

Bayescan v2.1 uses two alternative models with one including the effects of selection and one without. It then uses a reverse-jump Markov chain Monte Carlo to estimate the posterior probability of each model (Foll and Gaggiotti 2008). Parameters for *Bayescan v2.1* included 20 pilot runs of length 5K, 50K burn-in length, and a thinning interval of 10 with 5K final iterations. Prior odds for the neutral model was 10 and uniform prior on F_{is} had a lower bound of 0.0 and upper bound 1.0, with 1.0 representing complete inbreeding. Outlier loci (64) were selected using q-values ≥0.5 for substantial evidence of selection.

For *Bayenv2,* population allele frequencies of SNPs from reciprocal garden plants were used to generate a covariance matrix for populations to control for population

structure. Four separate covariance matrices were generated running the Markov chain Monte Carlo to 10^6 iterations and visualized to monitor chain convergence. Values of X^TX were empirically ranked and the top 1% of differentiated loci were conservatively retained as outliers (46 SNPs) (Guenther and Coop 2013). See details in Supplemental Methods. Finally, we compared outliers found in *Bayenv2* (Guenther and Coop 2013) with those outliers identified with *Bayescan. (STable 6).*

BayeScEnv (Villemereuil and Gaggiotti 2015) builds upon the methodology in *Bayescan* v2.1 by a reverse-jump Markov chain Monte Carlo to estimate the posterior probability of each model. However, in addition to the model with and without selection, it includes a third model where is linked to environmental variable. Outlier markers indicating significant signals of environment were identified as log q-value passing FDR significance threshold of 0.05.

For the pRDA analysis, three models were run: The full model (Model 1) considered both climate variables and geography as explanatory variables, Model 2 was a partial model in which geography explained the genetic data conditioned on climate variables, and Model 3 was a partial model in which climate variables explained genetic data conditioned on geography. All precipitation variables were used in the model except for precipitation of the driest year and number of precipitation events >1.25 cm (Table 1) due to collinearity.
